# Processing Verb Meanings and the Declarative/Procedural Model: A Developmental Study

**DOI:** 10.3389/fpsyg.2021.714523

**Published:** 2021-09-30

**Authors:** Nicolas Stefaniak, Véronique Baltazart, Christelle Declercq

**Affiliations:** Laboratoire C2S (Cognition, Santé, Société), Université de Reims Champagne-Ardenne, Reims, France

**Keywords:** procedural/declarative model, language acquisition, verb comprehension, language understanding, typicality effect, grammaticality judgment

## Abstract

According to the Declarative/Procedural Model, the lexicon depends on declarative memory while grammar relies on procedural memory. Furthermore, procedural memory underlies the sequential processing of language. Thus, this system is important for predicting the next item in a sentence. Verb processing represents a good candidate to test this assumption. Semantic representations of verbs include information about the protagonists in the situations they refer to. This semantic knowledge is acquired implicitly and used during verb processing, such that the processing of a verb preactivates its typical patients (e.g., *the window* for *break*). Thus, determining how the patient typicality effect appears during children’s cognitive development could provide evidence about the memory system that is dedicated to this effect. Two studies are presented in which French children aged 6–10 and adults made grammaticality judgments on 80 auditorily presented sentences. In Experiment 1, the verb was followed by a typical patient or by a less typical patient. In Experiment 2, grammatical sentences were constructed such that the verb was followed either by a typical patient or by a noun that could not be a patient of that verb. The typicality effect occurs in younger children and is interpreted in terms of developmental invariance. We suggest that this effect may depend on procedural memory, in line with studies that showed that meaning is necessary to allow procedural memory to learn the sequence of words in a sentence.

## Introduction

The Declarative/Procedural Model (D/P Model) describes how long-term memory systems, namely the declarative and procedural memory systems, contribute to language processing and learning (Ullman, 2001, 2020). Declarative memory is hypothesized to be involved in learning knowledge about facts and events, that is, semantic and episodic knowledge. Knowledge in this memory system is mainly acquired explicitly. Procedural memory, on the other hand, is involved in learning and processing motor and cognitive skills. Procedural memory is particularly involved in learning probabilistic sequential rules (Simor et al., 2019). Knowledge in the procedural memory system is mainly implicited (Squire, 1992). The two memory systems are believed to subserve language learning and processing in a complementary fashion. According to the D/P Model, declarative memory underlies the mental lexicon, which contains knowledge of words, whether idiosyncratic word-specific knowledge or memorized complex forms, whereas grammar (and other structural rules of language), which is a rule-governed system, is associated with procedural memory (Ullman, 2001, 2007). During language comprehension, as soon as each incoming word is encountered, its meaning is activated in memory and incorporated into the mental representation of the statement under construction (Kintsch, 1988, 2001). As a result, this representation is gradually adapted and refined, taking into account the meaning of the incoming word and the context. In the framework of the D/P Model, declarative memory is involved in the activation of meaning. On the other hand, procedural memory applies to grammar, and more specifically to grammatical-structure building. In other words, procedural memory is claimed to be important in the combination of words into complex hierarchical structures. Since procedural memory is supposed to be involved in predicting probabilistic outcomes, this system should be important for predicting the next word in a sentence, as proposed by Ullman (2020; see also Chang et al., 2006).

Verb processing represents a good candidate to test the assumption that word meaning activation is associated with declarative memory, while procedural memory is involved in the combination of words in complex sequences and prediction of the next word in a sentence. Research into verb semantics has shown that the semantic representations of verbs in memory encompass not only their meanings but also specific knowledge of the protagonists in the situations referred to McRae et al. (1997) and Ferretti et al. (2001). These authors’ experiments have shown that people possess specific knowledge about which kinds of words are typically involved in the action referred to by a transitive verb – specifically, the agents and patients – and they can verbalize this knowledge. For example, people know that *to arrest* has *cop* as a frequent agent and *crook* as a frequent patient. This knowledge is semantically linked to the verb’s core meaning, which commonly refers to an event or an action. Furthermore, McRae et al. (1997) showed that the sets of possible patients or agents of transitive verbs have a graded structure. More specifically, agents and patients have different degrees of typicality with respect to a verb. In other words, for a given verb, some entities are better agents or patients than others. For instance, the most typical patient of the verb *to accuse* is *suspect*. It is possible to assess the typicality of an agent or patient by asking participants to name the first one that comes to mind. In this situation, some are produced very frequently and thus can be considered as typical, whereas many others are mentioned less frequently and can be considered as moderately or weakly typical (Declercq and Le Ny, 2008). Le Ny and Franquart-Declercq (2002) assumed that this knowledge is involved in verb processing such that the processing of a verb preactivates the salient semantic features of its typical agents and patients. When a sentence is processed, this leads to expectations about the potential agent and/or patient that will be associated with the verb.

The study of verb processing during language comprehension raises some questions about the D/P Model that this study aims to address by focusing on the patient typicality effect. The first question concerns the sources of the gradient structure of possible patients. According to the D/P Model, the meanings of verbs are supposed to be accessed with declarative memory, but it also seems that several aspects of verb semantics might be acquired through implicit learning mechanisms. Indeed, in the extensive body of research concerning verb acquisition, many studies have explored how and when children acquire the grammatical category of verb, how they acquire verb-argument structure, and when they know which components of reality verbs refer to. There is convergent evidence that these concepts are acquired during the first years of life (Gentner, 1978; Gleitman, 1990; Lidz and Gleitman, 2004; Pulverman et al., 2006; Meints et al., 2008; Abbot-Smith and Tomasello, 2010). However, unlike the acquisition of verb semantics, little information is available concerning agent and patient typicality effects. Evidence from some studies suggest that children are sensitive to the typicality of agents and patients for action verbs between 18 and 24months of age (Poulin-Dubois et al., 2002; Serbin et al., 2002; Meints et al., 2008). More specifically, in those studies, children were surprised to see an individual performing an atypical action as an agent (e.g., they looked longer at a man putting on lipstick than at a woman) or acting on an atypical patient (e.g., a woman eating a houseplant rather than an apple). In addition, knowledge of nonlinguistic events has been shown to predict later verb comprehension (Konishi et al., 2016; Lakusta et al., 2017). Thus, individuals seem to learn the meanings of verbs, and more specifically the gradient structure of the sets of possible patients, thanks to the frequency with which protagonists take part in these events. Recent literature concerning the acquisition of meaning is consistent with this conclusion, but it also stresses the role of distributional information, a concept referring to the fact that words co-occur differentially in discourse. Two main sources are evoked to explain meaning acquisition: (1) sensory and motor experience with words’ referents in everyday life and (2) linguistic experience, that is, words’ verbal associations, co-occurrence in discourse, and syntactic information (Landauer and Dumais, 1997; Redington and Chater, 1997; Andrews and Vigliocco, 2010; Jamieson et al., 2020). Returning to verb meanings, this means that the gradient structure of sets of possible patients in memory is grounded in events, individuals have experienced and in discourse they have heard and/or read. From this perspective, the gradient structure of sets of possible patients is learned thanks to implicit learning mechanisms, and thus procedural memory is a good candidate for acquiring these abilities.

The role of implicit learning mechanisms in language acquisition has been examined for several decades in a framework that adopted a probabilistic and distributional approach to language use (e.g., Redington and Chater, 1997; Seidenberg and MacDonald, 1999). Research within this framework has highlighted the impact of statistical regularities of discourse on certain aspects of language knowledge (Redington and Chater, 1997; Redington et al., 1998; Seidenberg and MacDonald, 1999; Saffran, 2001). For instance, phonotactic regularities help children to segment speech into words (Brent and Cartwright, 1996; Saffran et al., 1996), and statistical regularities help them acquire the syntactic structure of the language (e.g., Redington et al., 1998; Thompson and Newport, 2007). It was only more recently that researcher began investigating the impact of statistical regularities on semantic aspects. Several studies have shown that both the regularity with which entities reliably co-occur in the world and the regularity with which their labels reliably co-occur in discourse contribute to the organization of preschoolers’ semantic knowledge, and specifically to the relationships between concepts (Matlen et al., 2015; Unger et al., 2020a,b). In addition, these regularities are exploited when both children and adults acquire word meanings (Chen et al., 2011; Leung and Williams, 2011; Paciorek and Williams, 2015; Wojcik and Saffran, 2015; Li et al., 2020). Paciorek and Williams (2015) specifically investigated implicit learning of the semantic preferences of novel verbs, defined as the tendency of a word to co-occur with words having similar semantic features. Participants were first given a task requiring them to learn the meanings of novel verbs, which they could infer from the context. However, there was also a hidden regularity corresponding to a semantic preference rule such that half the instances of each kind of verb co-occurred only with abstract nouns while the other half co-occurred only with concrete nouns. In the subsequent testing phase, participants were required to judge whether pairs of novel verbs and nouns could appear together in a phrase. The results indicated that participants responded positively more often when the pairs were consistent with the semantic preference rule, even when they reported that they were unaware of such a rule. These data suggest that participants had implicitly acquired the verbs’ semantic preferences. Our study aims to show that such implicit knowledge of verb structure plays a role during language comprehension. This leads to a second question concerning the D/P Model.

As noted above, while semantic knowledge is associated with declarative memory, the combination of words is associated with procedural memory, which should be involved in predicting the next word in a sentence; the DP Model postulates that this is especially true from the perspective of grammatical-structure building. Yet understanding sentences involves combining meanings that are supposed to be stored in declarative memory. In addition, an impressive body of research has addressed the question of prediction during language comprehension, including by focusing on semantic information. Indeed, researchers have shown that contextual information facilitates the semantic processing of incoming words (Kuperberg and Jaeger, 2016). This facilitation is explained by a predictive preactivation of upcoming information thanks to contextual information. Thus, there is a need to clarify the roles of the declarative and procedural memory systems during language comprehension. A solution may lie in the interaction of the two systems, as Ullman (2020) outlined. This idea is consistent with the dual-path model (Chang et al., 2006), which was proposed to describe sentence production but can be extended to sentence comprehension. This model considers that prediction occurs during language processing in that upcoming words are predicted, using the meaning of the immediately preceding word as input. This conceptualization is similar to the D/P Model since two pathways are described: a meaning system and a sequencing system. In other words, the functions attributed to these two systems are similar to the roles attributed to declarative and procedural memory, respectively. Interestingly, the dual-path model adds to the D/P Model since it considers how the systems work together. The sequencing system implicitly learns the syntactic structure of language but, in so doing, it also acquires semantic information. Specifically, it learns the thematic roles of verbs and semantic information concerning their agents and patients and can predict what words are allowed in the position N+1. For instance, the sequencing system learns what kinds of entities can be arrested or accused and predicts that the verbs *arrested* or *accused* should be followed by a word referring to a human being rather than an inanimate object. Similar frameworks have been proposed in the field of language acquisition (Alishahi and Stevenson, 2010). Thus, the dual-path model could explain how semantic knowledge can contribute to processing, which is assumed to depend on procedural memory. Our view of the role of procedural memory agrees with an idea of Chang et al. (2006) that procedural memory determines which kinds of patients are allowed after a given verb and which are not.

The purpose of this study is to show that semantic knowledge contributes, in both children and adults, to the kind of processing that is supposed to rely on procedural memory. More specifically, we studied the patient typicality effect from a developmental perspective. Given that, according to Kail (2004), the study of sentence structures has been successfully investigated with different methods from the age of 6, and that metalinguistic abilities are acquired during the middle childhood years (e.g., Gombert, 1990), we have chosen to investigate the age range from 6 to 10years. To this end, we used a grammaticality judgment task, which is commonly used in the study of language acquisition, in both typical development and developmental language disorders (van der Lely and Ullman, 1996; Rice et al., 1999; Kail, 2004; Tremblay, 2005). In this task, participants are asked to decide whether a sentence is “correct” or “well-formed” in their language. These judgments are meant to provide information about an individual’s grammatical competence. Thus, performing this task relies mainly on procedural memory, as described in the D/P Model. In our study, the participants listened to grammatical and ungrammatical sentences (the latter were taken from Kail’s materials) and were asked to decide whether or not the sentences were correct. We focused on grammatical sentences that varied according to patient typicality. These patients were rated according to free association norms obtained in adults. We hypothesized that grammaticality judgments would be affected by the typicality effect, even in children. More specifically, we expected shorter reaction times (RTs) and greater accuracy when a verb was associated with a typical patient. In Experiment 1, the grammatical sentences were constructed such that the verb was followed by a typical patient or by a less typical patient. In this condition, both types of sentences were easily understood because the meaning was obvious. In Experiment 2, the grammatical sentences were constructed such that the verb was followed either by a typical patient or by a noun that could not be a patient of that verb (unusual patient), thus producing a meaningless sentence. For instance, in the sentence “Sometimes, when he goes for a walk in the countryside, the walker forgets…” (*Parfois, quand il part se promener dans la campagne, le promeneur oublie…*), the verb was followed either by the typical patient “his key” (*sa clé*) or by “his mud” (*sa boue*), which is not a typical patient of this verb. In this experiment, the difference between a typical patient and an unusual one resides in their semantic relation with the verb. The possibility cannot be excluded that sentences with unusual patients would be considered as incorrect, particularly during childhood, since they sound funny.

We might hypothesize that, although the typicality effect helps language comprehension, it depends on procedural memory and becomes efficient as early as age 6. Nevertheless, another view could be that this effect (also) depends on declarative memory. Indeed, when a verb is activated, some of its activation is thought to spread to its most typical patients due to the spreading activation process (Kintsch, 2001; Drouillet et al., 2018). The developmental invariance hypothesis can help to delineate between these two memory systems. Developmental invariance means that a specific ability reaches adult level early in life. In the context of the D/P model, procedural memory should be developmentally invariant, while declarative memory should increase with age. Procedural memory becomes efficient early during development (Canfield and Haith, 1991; Meulemans et al., 1998; Amso and Davidow, 2012; Ullman, 2020); Even if the results concerning declarative memory are less clear (Perruchet et al., 1995), most studies suggest a developmental path (e.g., Bauer, 2008; Lum et al., 2010). Thus, if the patient typicality effect depends on declarative memory, the typicality effect (i.e., the difference between the highly typical and the less typical/unusual patients) should increase with semantic richness which grows with age. Although semantic richness may depend on implicit learning mechanisms (Rabovsky et al., 2012), adults should have more semantic richness because they have been exposed to language for longer. Conversely, if procedural memory determines what kinds of words are allowed as patients for a given verb, we can hypothesize that the process is developmentally invariant (Meulemans et al., 1998; Lum et al., 2010) which means that the difference between highly typical and less typical/unusual patient should be quite similar in all age groups. More specifically, we make two predictions. First, given that declarative memory (e.g., Bauer, 2008; Lum et al., 2010), semantic richness (Krethlow et al., 2020), and metalinguistic knowledge (Cairns et al., 2006) increase with age, we should observe an increase in accuracy and a decrease in reaction times with age. Second, if the interaction between age and the typicality of the patient is significant, it would mean that declarative memory is a better candidate for explaining the patient typicality effect; conversely, if we have evidence in favor of the absence of interaction, we believe that procedural memory should be a better candidate for explaining this effect. Rumelhart and Levin (1975) modeled that the typicality effect of the patient could depend on spreading activation and we know that semantic priming increases with age (e.g., Girbau and Schwartz, 2011). We should observe that the difference between typical and less typical patients increase with age, and so should observe an interaction between age and condition. Conversely, Bauer (2008) argues that, because procedural memory is underlain by structures that mature first, it must reach adult-like levels early. Thus, the difference between typical and less typical/unusual patients must be quite similar among age groups. Moreover, according to Skeide et al. (2014), children do not process semantics independently from syntax, which allows us to form an alternative hypothesis: If the typicality effect depends on procedural memory, children would more likely consider the sentence with an unusual patient as ungrammatical since they are not able to discriminate a semantic violation from a grammatical one.

In the framework of the D/P Model, we can make the following predictions. First, according to the developmental invariance hypothesis, if the typicality effect is acquired through implicit procedural memory mechanisms, we should observe it in all age groups and no interaction between age and typicality effect should be observed. Conversely, declarative memory would be a better candidate to explain the typicality effect for verbs if developmental stages in the effect are observed. Moreover, if both systems contribute to the typicality effect, as can also be hypothesized, sentence processing should be more disturbed by unusual patients (e.g., syntactically compatible, but meaningless): procedural memory should determine which patients are allowed, while declarative memory should play a role in the understanding of the sentence. An unusual patient is syntactically compatible, but no meaning can be found. Both systems should then determine whether a meaning can be constructed for this new utterance. When this is the case, for instance with new metaphors (Drouillet et al., 2018), both declarative and procedural memory must be updated: the former to create a relevant meaning and the latter to update the kinds of patients that are allowed. When the patient is unusual, no meaning can be generated, and the allowable patients for the presented verb cannot be updated in procedural memory. The decision that should be made is that the sentence is not grammatical since procedural memory does not allow that patient for that verb. These predictions follow from the dual-path model of Chang et al. (2006). For the D/P Model, it would mean that procedural memory is not only involved in the grammatical, rule-governed aspects of language but also in meaning construction.

## Experiment 1

Experiment 1 was designed to test the patient typicality effect. We contrasted sentences that ended with a highly typical patient or a less typical patient. We tested whether the typicality effect depended on age by comparing 6-, 8-, and 10-year-old children with adults.

### Method

#### Participants

Ninety-three participants took part in the study. The participants were 6-, 8-, and 10-year-old children and adults. All participants were native speakers of French. The participants’ characteristics are presented in Table 1. This study was conducted in accordance with the declaration of Helsinki. Before the beginning of the study, each child’s parents and the children themselves gave their informed consent to take part in the study.

**Table 1:** Summary of participants’ characteristics in Experiment 1.

	6-year-old	8-year-old	10-year-old	Adults
Sex (F/M)	22 (6/16)	21 (7/14)	27 (15/12)	23 (18/5)
Mean age in months (minimum–maximum)	76.79 (72–83)	100.40 (94–107)	124.18 (118–131)	264.05 (211–484)

We asked parents to complete a self-report questionnaire to exclude participants who were suffering from neurodevelopmental disorders according to the DSM-5 criteria (American Psychiatric Association, 2013). Children for whom problems were reported at birth or who were taking drugs that could alter cognitive processing were also excluded. Fourteen participants were excluded for one or more of these criteria: four in the 6-year-old group, two in the 8-year-old group, four in the 10-year-old group, and four in the adult group.

Finally, we also ensured that the participants’ hearing was unimpaired or corrected with Eartest software (Wallroth, 2007). Each ear was tested separately at 20dB with the following frequencies: 500, 1,000, and 2,000Hz.

#### Material

The experimental material was constructed in several steps. In the first phase, 80 verbs were presented to 244 adults, who were asked to give two possible patients for each one. More specifically, they were asked to identify two words that can undergo the action evoked by the verb. For instance, they were asked to list two things that can be watered. The selection criterion was that the association between the patient and the verb had to be provided with a frequency greater than 0.2, which means that, for a selected patient, at least 49 individuals (244×0.2) had provided this patient among the two patients that they were asked to provide. This criterion allowed us to select 26 verbs and their typical patients (e.g., *build – house*; *construire – maison*). The less typical patients were selected using a two-step procedure. First, we selected potential less typical patients from patients that were paired for the number of syllables with the typical patients and for which the production frequency was as low as possible (<0.05). When it was not possible to match the typical and less typical patients for number of syllables, the word that best matched the number of syllables was chosen. After this first step, we matched typical and less typical patients for lexical frequency. Word frequencies were obtained from the MANULEX database (Lété et al., 2004), which provides word frequencies for children from 6 to 11years old. If one of the two patients was not found in the MANULEX database, the words were searched for in LEXIQUE.org (New et al., 2001). In other words, for each typical–less typical pair of patients, data from the MANULEX database were used as much as possible. Only if no pairing was possible in the MANULEX database did we use LEXIQUE.org. In this case, among the different possible choices, the word selected was the shortest and most frequent one. We fulfilled these criteria for 18 of the initial 80 verbs (for detailed material, see Appendix 1). For instance, the typical patient for the verb *to water* was *flowers*, and the less typical patient was *sister*. For these 18 items, the less typical patient was found in the MANULEX database. The multivariate analysis of variance revealed that patients in each condition differed neither in frequency nor in number of syllables [Pillai’s Trace (2,33)=0.024, *p*=0.625].

Then, the sentences were created. These sentences were constructed as follows: one or two adjuncts followed by the canonical order in French (i.e., SVO). One of these sentences was: *Ce matin, tandis que les nuages s’éloignent dans le ciel, le soleil éclaire la pièce/le livre* (“This morning, as the clouds vanish from the sky, the sun lights up the room/the book”). The contextual information was chosen so that the verb-patient associations could be used with the same sentence in both conditions (for a detailed presentation of the material, see Appendix 2). To ensure that a given patient was not more predictable from the beginning of the sentence in one condition than in the other, we measured the semantic similarity of the beginning of the sentence and the patient using Latent Semantic Analysis (Foltz et al., 1998). This analysis revealed that the semantic similarity for the typical (*M*=0.344, *SD*=0.10) and less typical (*M*=0.373, *SD*=0.08) patients did not differ, *t*(17)=1.00, *p*=0.331.

Finally, these 36 sentences (18 in the typical and 18 in the less typical condition) were complemented by 36 sentences in which a grammatical violation was introduced, for a total of 72 sentences. The 36 ungrammatical sentences were inspired by Kail (2004) and had the same form as the grammatical sentences. For instance, one sentence in which the grammatical violation was an article-subject inversion was: *Sur l’île noire, après avoir coulé le bateau, bandit le enfouit le trésor* (“On the black island, after sinking the boat, bandit the buries the treasure”). The nature of the grammatical violations could be either an inversion or an agreement error. The grammatical violation could be situated at any of the following locations: article–subject, article–patient, subject–verb, or verb–patient. Thus, contrary to a study of Kail (2004), the location of the violation was not predictable to prevent participants from anticipating a possible violation. These grammatical violations do not correspond to usual violations that are made in everyday speech. The material (including Experiment 2) is presented in Appendix 2.

#### Procedure

Participants had to perform a grammatical judgment task. They were given the following instructions: “You’re going to hear some sentences. Listen to them very carefully. For each of the sentences that you are going to hear, I would like you to decide whether it is correct or not; that is, decide whether or not it has good grammar.” For instance, tell me if, in your opinion, the sentence that I am going to say has good grammar or not: *Après s’être garé, les lunettes sur les yeux,* and *le chauffeur examine carte la* (“After parking the car, wearing his glasses, the driver examined map the”). After the child’s response, the experimenter said, “You’re right, we should say that the driver examines the map. Here is another example: *En rentrant chez lui, avant de faire ses devoirs,* and *l’école mange le goûter* (“When it comes home, before doing its homework, and the school eats a snack”). You are right, the sentence is unusual and it is difficult to understand what it means, but the grammar is correct. Do not forget that you have to decide whether the sentences have good grammar – whether they are correct or not. You must answer as quickly and as accurately as possible. If the sentence is correct, press the green key; if the sentence is incorrect, press the red key. Before beginning the task, you will be allowed to practice. Do you have any questions?” If the child did not have any questions, or after any questions had been answered, the experimenter told the child, “Now, we are going to start.” Then, 80 sentences were orally presented. The first eight sentences were practice examples and the other 72 were the critical items. After each sentence, a blank screen was presented until the participant’s response. The next item was presented directly after the participant’s response.

The sentences were recorded by a female native speaker of French using a neutral voice. Sentences were equalized for their duration and intensity using Audacity®. They were presented aloud to the children through headphones, using E-prime 1.2® software. Responses and latencies (from the end of the sentence) were recorded. The sentences were randomly presented with a 2-s interval between the response and the next sentence. There was no time limit to respond. The experiment lasted about 20min with a break after 36 sentences.

All data and material are available at https://osf.io/4znu9/.

### Results and Discussion

The analyses compared the accuracy (proportions of correct responses) and RTs as a function of type of item and age group. Analyses were performed with R (R Core Team, 2021). Preprocessing was done with the stringr (Wickham, 2019), reshape (Wickham, 2007), and dplyr (Wickham et al., 2020) packages. We used the lme4 package (Bates et al., 2015) for the linear mixed models, and we complemented the results with functions in MuMIn (Barton, 2020) and lmerTest (Kuznetsova et al., 2017). Bayes factors were obtained with the bayestestR package (Makowski et al., 2019). Finally, contrasts were done with the emmeans package (Lenth, 2020) and plots with the emmeans (Lenth, 2020) and lattices (Deepayan, 2008) packages. The analyses investigated accuracy and RTs. For RTs, analyses were performed on correct responses only.

#### Accuracy

To ensure that all participants (especially the younger ones) had a sufficient understanding of the task, we performed one-sample *t*-tests by age group on the number of correct responses (i.e., the sum of grammatical responses in the highly typical and less typical conditions, and ungrammatical responses for items in which there was a grammatical violation). The true mean was set at the chance level (i.e., 36 out of 72). These analyses revealed that the number of correct responses was above the chance level for all age groups. Indeed, the mean correct responses were 42.00 [*t*(17)=3.34, *p*=0.004, 95% CI=38.02–45.79], 52.63 [*t*(18)=6.72, *p*=0.007, 95% CI=47.43–57.83], 61.35 [*t*(22)=15.33, *p*<0.001, 95% CI=57.92–64.78], and 66.68 [*t*(18)=22.26, *p*<0.001, 95% CI=63.78–69.58] for the 6-, 8-, and 10-year-old and the adults, respectively. These effects remained significant even after Holm’s correction.

To assess accuracy, we performed generalized mixed model estimation with a binomial distribution. Random effects were participants and items. Random effects for items and participants are presented in Appendix 3. Then, we tested the improvement in Likelihood Ratio Test (LRT) for the following fixed effects: Type of item (two levels: less typical vs. highly typical) as a within-participant variable, Age group (four levels: 6-, 8-, and 10-year-old, and adults) as a between-participants variable and the Type of item×Age group interaction. The two main effects were significant, but the interaction was not significant. These models are summarized in Table 2.

**Table 2:** Results of the generalized mixed model on accuracy in Experiment 1.

	LRT^*^ (df)	*p* value	Pseudo-*R*^2^	Δ pseudo-*R*^2^^**^
Random effect			0.368	0.368
Type of item	9.47 (3)	0.024	0.372	0.004
Age group	77.78 (3)	<0.001	0.403	0.031
Type of item×Age group	1.66 (3)	0.645	0.404	<0.001

Likelihood ratio test (and degrees of freedom), *p* value and Nagelkerke pseudo-*R*^2^ for Type of item (highly typical vs. less typical), Age group, and the interaction between Type of item and Age group on accuracy.

* LRT, likelihood ratio test.

** Δ pseudo-*R*^2^ is the difference between pseudo-*R*^2^ between models, which provides the effect size for each specific effect.

**Figure 1:**
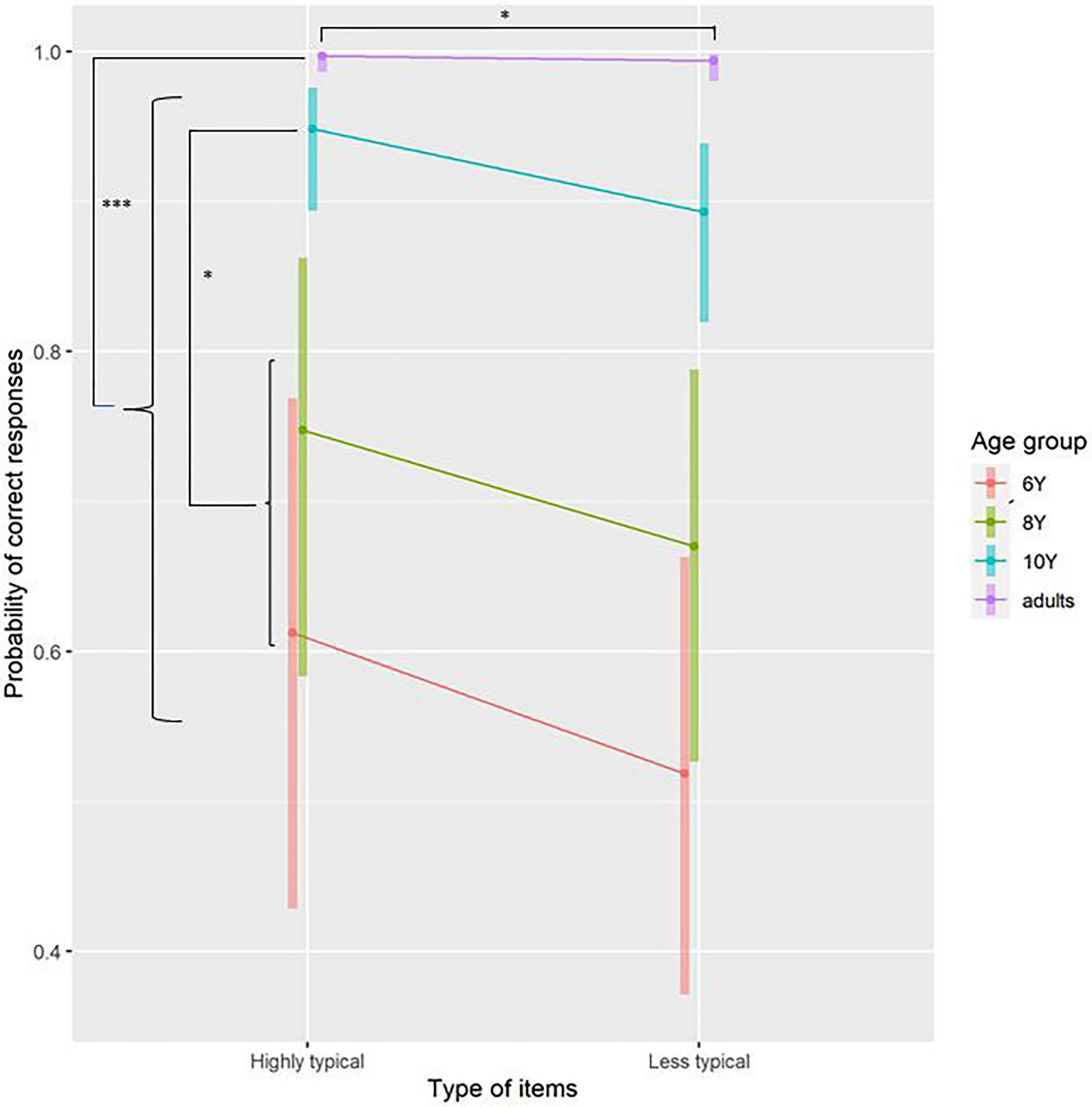
Probability of correct responses in Experiment 1 plotted separately for Age group and Type of item. Error bars are 95% CI. ^*^p<0.05 and ^***^p<0.001.

The analyses revealed that highly typical patients were processed more accurately than less typical patients. To further explore the differences between age groups, we compared adults with children, then 10-year-old with the combination of both 6- and 8-year-old, and finally the 8-year-old with the 6-year-old. Given that the interaction term was not significant, we did not further explore the contrasts for the interaction. These results are presented in Table 3 and Figure 1, and mean and SE values are presented in Appendix 4.

**Table 3:** Contrasts following the generalized mixed model on accuracy in Experiment 1 for Type of item and Age group.

Contrast	Estimate	*z*	*p* value	OR (95% CI)
Less typical – Highly typical	0.28	1.91	0.056	1.32 (0.99–1.75)
Children – Adults	−1.05	7.41	<0.001	0.35 (0.27–0.46)
6 & 8–10	−0.65	5.18	0.016	0.53 (0.41–0.67)
6–8	−0.32	1.45	0.42	0.73 (0.48–1.12)

The first contrast compares accuracy between highly and less typical items. The second, third, and fourth contrast compare differences in accuracies between age groups, i.e., adults vs. children, 10-year-old vs. younger ones, and 6-year-old vs. 8-year-old. Estimate is the log of difference, *z* is the statistic, and OR is the odds ratio, which is the effect size for the contrast.

Although it is noteworthy that the contrast between less typical and highly typical patients was not significant, in the main analysis, both the Akaike information criterion and the difference between deviances suggest that the main effect of Type of item was significant. This difference can be explained by the sequential nature of the estimation when we compared the different models, whereas contrasts were performed on the final model. Thus, we believe that it makes sense to compute the contrast on the difference between less typical and highly typical items so that we can determine the odds ratio. The results revealed that errors were 1.27 times more probable for less typical than for highly typical items. Moreover, the probability of making a mistake was 3.45 times lower (i.e., 1/0.29) for adults than for children, and 1.49 times lower (i.e., 1/0.67) for 10-year-old children than younger ones. However, 6- and 8-year-old children did not have different accuracy rates.

#### Reaction Times

Analyses were performed on correct responses only. We performed linear mixed model estimation for RTs. Random effects were participants and items. To meet the assumptions (i.e., normality) and eliminate especially long RTs (i.e., up to 42s), we removed outlier observations using Grubbs’ test on residuals of the model with random effects only. Ninety observations (i.e., 4.2% of observations) were considered to be outliers.

Both random effects (items and participants) were significant (*p*<0.001); together, they explained 63% of the variance. Then, we tested the improvement in LRT for the following fixed effects: Type of item (two levels: less typical vs. highly typical) as a within-participant variable, Age group (four levels: 6-, 8-, and 10-year-old, and adults) and the Type of item×Age group interaction. The effect of Age group was significant while the other two effects were not. These models are summarized in Table 4.

**Table 4:** Results of the linear mixed model on reaction times (RTs) in Experiment 1.

	LRT^*^ (df)	*p* value	Pseudo-*R*^2^	Δ pseudo-*R*^2^^**^
Random effect			0.631	
Type of item	0.79 (3)	0.852	0.632	<0.001
Age group	31.04 (3)	<0.001	0.637	0.006
Type of item×Age group	0.10 (3)	>0.99	0.637	<0.001

Likelihood ratio test (and degrees of freedom), *p*-value and Nagelkerke pseudo-*R*^2^, delta of *R*^2^ between models (Δ pseudo-*R*^2^) for the effects of Type of item (Highly vs. Less typical items), Age group, and the interaction between Type of item and Age group on RTs.

* LRT=Likelihood ratio test.

** Δ pseudo-*R*^2^ is the difference between pseudo-*R*^2^ between models, which provides the effect size for each specific effect.

**Figure 2:**
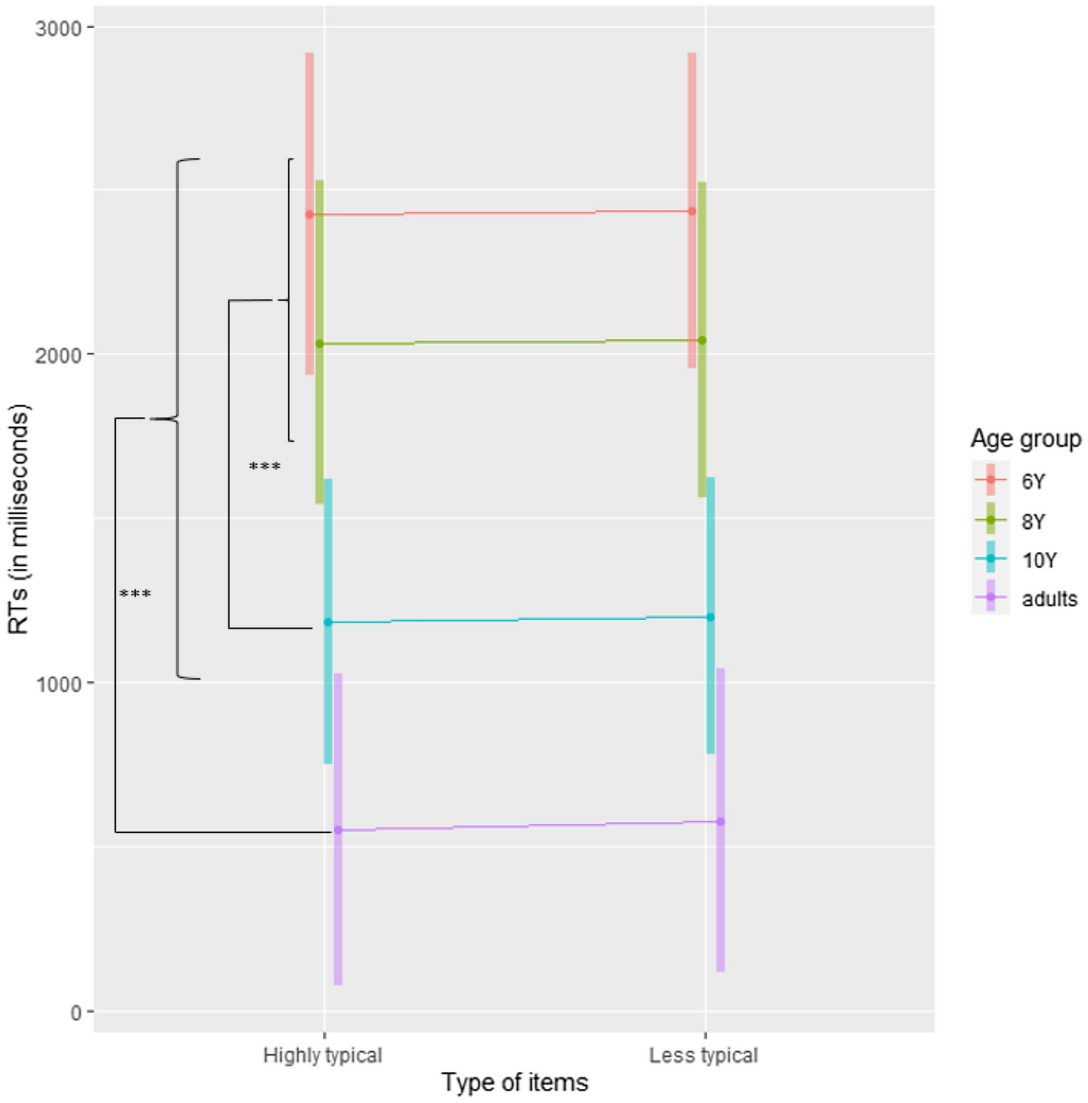
Mean reaction times in Experiment 1 plotted separately for Age group and Type of item. Error bars are 95% CI. ^***^p<0.001.

To further explore the differences between Age groups, we performed Helmert planned contrasts: we compared adults with children, then 10-year-old with the combination of both 6- and 8-year-old, and finally the 8-year-old with the 6-year-old. Given that the effect of Type of item and the interaction were not significant, no contrast was performed for these terms. For all contrasts, Satterthwaite’s method was used to estimate degrees of freedom. The results of the contrasts are summarized in Table 5. These results are presented in Figure 2, and means and SEs are presented in Appendix 5. Moreover, the classical ANOVA table with Satterthwaite’s method for the estimation of the degrees of freedom is presented in Appendix 6.

**Table 5:** Contrasts among age groups on RTs in Experiment 1.

Contrast	Estimate	*t* (df)	*p* value	Cohen’s *d* (95% CI)
Children – Adults	330.5	4.93 (71.6)	<0.001	0.29 (0.16 – 0.41)
6 and 8–10	346.9	3.83 (72.2)	<0.001	0.30 (0.14–0.46)
6–8	196.8	1.16 (73.3)	0.25	0.17 (−0.12 – 0.46)

The estimate is the mean difference between the groups, *t*(df) is the statistic, and Cohen’s *d* is the effect size.

These contrasts revealed that adults responded faster than children, 10-year-old children responded faster than younger ones, and there was no difference between 6- and 8-year-old children.

Together, these results showed that there is a small patient typicality effect that appears only in accuracy results but not RTs. The fact that the interaction was not significant suggests that the typicality effect is present as early as age 6.

## Experiment 2

The aim of Experiment 2 was to determine how children processed unusual patients. More specifically, we wondered whether the patient typicality effect would be observed with unusual patients. This experiment might also provide information about the impact of procedural memory on meaning processing in language. Indeed, if participants consider sentences in which the patients were meaningless in the context of the sentence to be ungrammatical (even though the grammar is correct), it could be interpreted as indicating that procedural memory intervenes to determine the legitimacy of the patient and, consequently, contributes to understanding semantics. It would play the role of a regulator that determines which words stored in declarative memory are or are not allowed.

### Method

#### Participants

Eighty-nine participants took part in the study. The inclusion and exclusion criteria were the same as in Experiment 1. The characteristics of participants in Experiment 2 are presented in Table 6.

**Table 6:** Summary of participants’ characteristics in Experiment 2.

	6-year-old	8-year-old	10-year-old	Adults
Sex (F/M)	20 (9/11)	27 (16/11)	20 (9/11)	22 (20/2)
Mean age in months (minimum–maximum)	75.52 (70–82)	98.26 (94–107)	123.54 (118–131)	256.34 (220–365)

Ten participants did not meet the inclusion criteria: one 6-year-old, two 8-year-old, three 10-year-old, and four adults.

#### Material and Procedure

In Experiment 2, the material and procedure were the same as in Experiment 1, except that the less typical patient was replaced by an unusual patient in the context of the sentence [e.g., *Ce matin, tandis que les nuages s’éloignent dans le ciel,* and *le soleil éclaire la pièce/la fonction* (“This morning, as the clouds vanish from the sky, the sun lights up the room/the function”), with typical and unusual patients, respectively]. An unusual patient was a patient that was never produced during the first phase. This means that none of the 244 adults produced this patient. The pairing procedure was the same as the one used in Experiment 1. Multivariate ANOVA for the pairing was presented in the method section of Experiment 1.

### Results and Discussion

The same analyses as in Experiment 1 were conducted in Experiment 2.

#### Accuracy

To ensure that all participants (especially the younger ones) had a sufficient understanding of the task, we performed one-sample *t*-tests by age group on the number of correct responses (i.e., the sum of grammatical responses in the highly typical and unusual conditions, and ungrammatical responses for items in which there was a grammatical violation). The true mean was set at the chance level (i.e., 36 out of 72). These analyses revealed that the number of correct responses was above the chance level for all age groups. The mean correct responses were 43.10 [*t*(18)=4.37, *p*=0.003, 95% CI=36.69–46.52], 48.76 [*t*(24)=7.82, *p*<0.001, 95% CI=45.39–52.13], 61.35 [*t*(16)=9.77, *p*<0.001, 95% CI=52.35–61.41], and 65.83 [*t*(17)=19.00, *p*<0.001, 95% CI=62.52–69.14] for the 6-, 8-, and 10-year-old and the adults, respectively. These effects remained significant even after Holm’s correction.

Moreover, given that the *unusual* condition might be especially difficult, we also performed one-sample *t*-tests on the number of correct responses in this condition only. In this case, the chance level is 9 out of 18. It appears that children did not respond above the chance level, with means of 7.26 [*t*(18)=1.48, *p*=0.157, 95% CI=4.79–9.74], 7.88 [*t*(24)=1.56, *p*=0.133, 95% CI=6.39–9.37], and 10.11 [*t*(16)=0.81, *p*=0.431, 95% CI=7.19–13.05] for the 6-, 8-, and 10-year-old, respectively. On the contrary, adults responded significantly above the chance level, with a mean of 16.22 [*t*(17)=9.49, *p*<0.001, 95% *IC*=14.62–17.83].

These results suggest that all the participants were able to perform the grammatical judgment task, but that children were not able to distinguish sentences in which the grammar is correct but no meaning can be found from sentences in which grammar is violated. We performed generalized mixed model estimation with a binomial distribution. Random effects were participants and items. Random effects for items and participants are presented in Appendix 7. We tested the improvement in LRT for the following fixed effects: Type of item (two levels: Unusual *vs.* highly typical) as a within-participant variable, Age group (four levels: 6-, 8-, 10-year-old, and adults) as a between-participant variable and the Type of item×Age group interaction. The two main effects were significant, but the interaction was not. These models are summarized in Table 7.

**Table 7:** Results of the generalized mixed model on accuracy in Experiment 2.

	LRT (df)	*p* value	Pseudo-*R*^2^	Δ pseudo-*R*^2^
Random effect			0.331	0.331
Type of item	155.31 (3)	<0.001	0.389	0.06
Age group	66.38 (3)	<0.001	0.413	0.024
Type of item×Age group	0.10 (3)	>0.99	0.413	<0.001

Likelihood ratio test (and degrees of freedom), *p* value, and Nagelkerke pseudo-*R*^2^ for the effects of Type of item, Age group, and the interaction between Type of item and Age group on accuracy.

**Figure 3:**
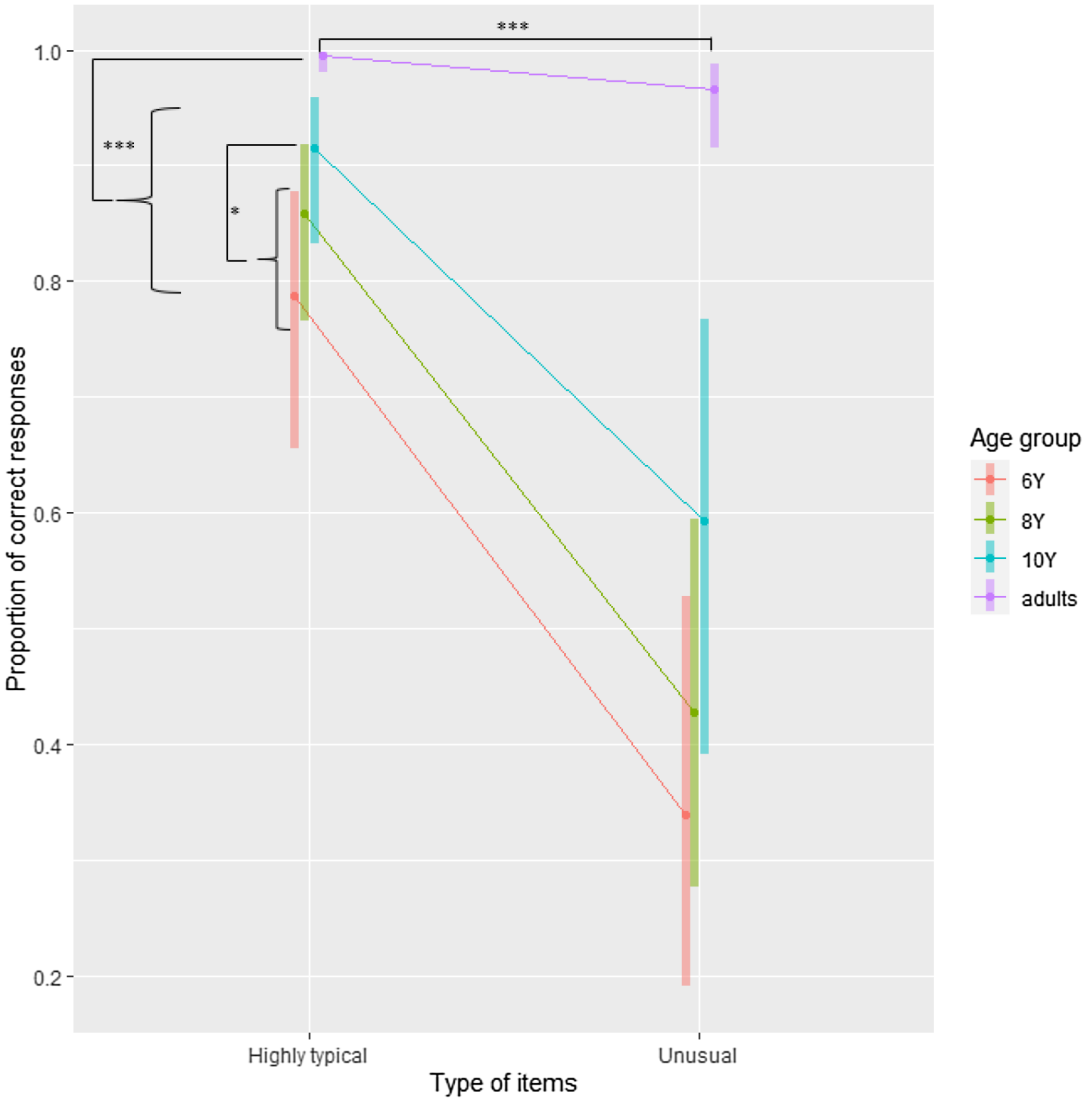
Probability of correct responses in Experiment 2 plotted separately for Age group and Type of item. Error bars are 95% CI. ^*^p<0.05 and ^***^p<0.001.

The analyses revealed that highly typical patients were processed more accurately than unusual patients. To further explore the differences between age groups, we compared adults with children, then 10-year-old with the combination of both 6- and 8-year-old, and finally the 8-year-old with the 6-year-old. Given that the interaction term was not significant, we did not further explore the contrasts for the interaction. Contrasts are presented in Table 8. These results are presented in Figure 3, and raw values are presented in Appendix 8.

**Table 8:** Contrasts following the generalized mixed model on accuracy in Experiment 2 for Type of item and Age group.

Contrast	estimate	*z*	*p*-value	OR (95% CI)
Unusual – Highly typical	0.99	6.11	<0.001	2.69 (1.96–3.70)
Children – Adults	−0.87	7.71	<0.001	0.42 (0.34–0.52)
6 and 8–10	−0.27	2.38	0.018	0.78 (0.60–0.95)
6–8	−0.22	1.20	0.231	0.80 (0.56–1.15)

The first contrast compares accuracy between highly typical and unusual items. The second, third, and fourth contrast compare differences in accuracy between age groups, i.e., adults vs. children, 10-year-old vs. younger ones, and 6-year-old vs. 8-year-old. Estimate is the log of difference, *z* is the statistics, and OR is the odds ratio, which is the effect size for the contrast.

These contrasts revealed that making an error was 2.44 times more probable when the item was unusual than when it was highly typical. Moreover, it was 2.56 times less probable (i.e., 1/0.39) for an adult to make a mistake than for a child to do so. However, the probabilities of correct responses for grammatical items were similar for all groups of children. Children in all age groups tended to consider unusual patients as ungrammatical, given that the percentage of correct responses is lower than 50%.

#### Reaction Times

Analyses were performed on correct responses only. We performed linear mixed model estimation for RTs. Random effects were participants and items. To meet the assumptions (i.e., normality) and eliminate especially long RTs (i.e., up to 65s), we removed outlier observations using Grubbs’ test on residuals of the model with random effects only. Seventy-two observations (i.e., 3.7%) were considered outliers.

Both random effects were significant (*p*<0.001); together, they explained 41% of the variance. We then tested the improvement in LRT for the following fixed effects: Type of item (two levels: less typical vs. highly typical) as a within-participant variable, Age group (four levels: 6-, 8-, 10-year-old, and adults) and the Type of item×Age group interaction. The effects of Age group and Type of item were significant while the interaction was not significant. These models are summarized in Table 9.

**Table 9:** Results of the linear mixed model on RTs in Experiment 2.

	LRT (df)	*p* value	Pseudo-*R*^2^	Δ pseudo-*R*^2^
Random effect			0.414	0.414
Type of item	92.53 (3)	<0.001	0.442	0.028
Age group	29.89 (3)	<0.001	0.451	0.009
Type of item×Age group	4.82 (3)	0.19	0.452	0.001

Likelihood ratio test (and degrees of freedom), *p* value and Nagelkerke pseudo-*R*^2^, delta of *R*^2^ between models (Δ pseudo-*R*^2^) for the effects of Type of item (Unusual vs. Highly typical items), Age group, and the interaction between Type of item and Age group on RTs.

**Figure 4:**
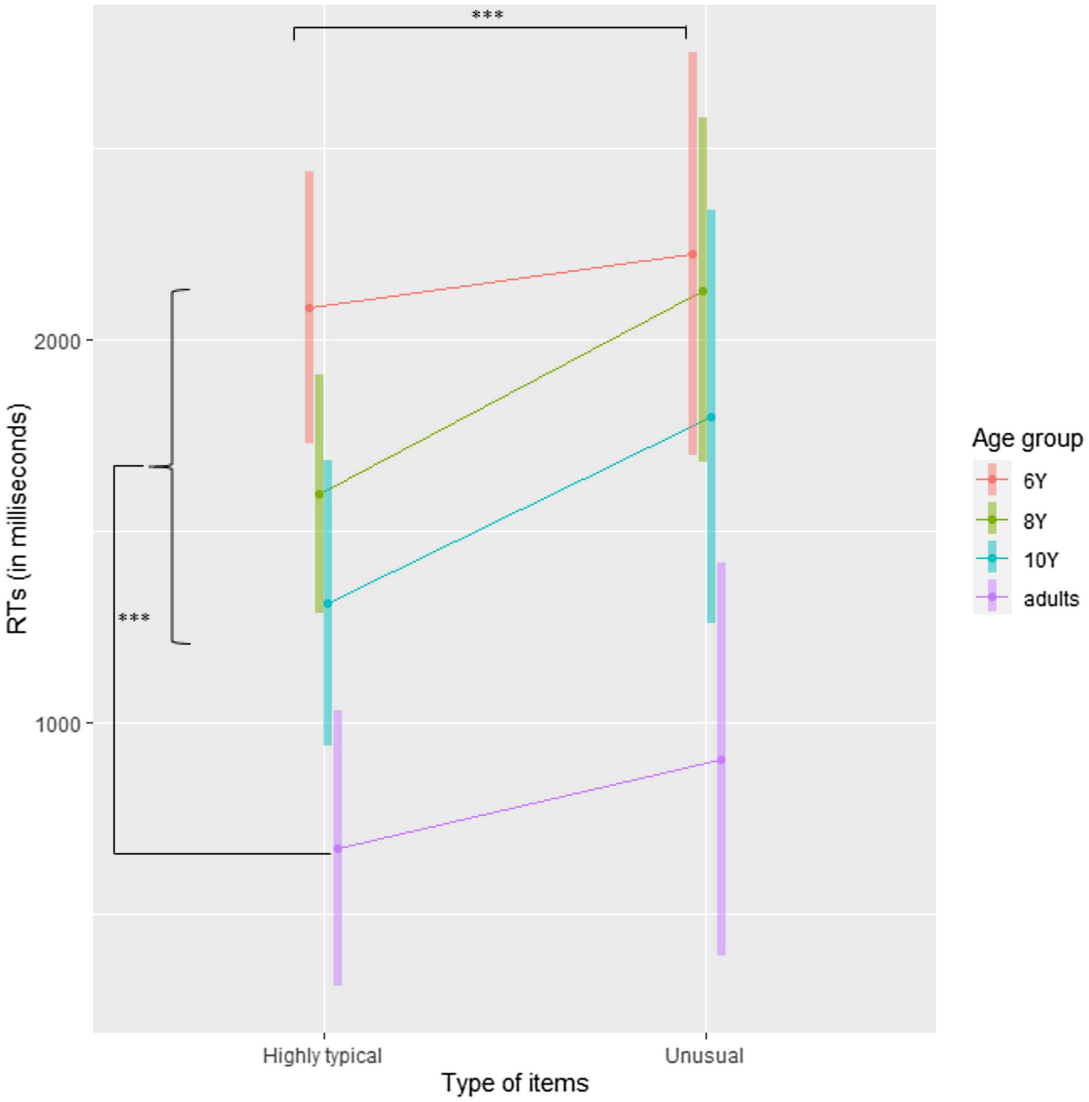
Mean reaction times in Experiment 2 plotted separately for Age group and Type of item. Error bars are 95% CI. ^***^p<0.001.

To further explore the differences in Age groups, we performed Helmert planned comparisons: we compared adults with children, then 10-year-old with the combination of both 6- and 8-year-old, and finally 8-year-old with 6-year-old. Given that the effect of Type of item and the interaction were not significant, no contrast was performed for these terms. For all contrasts, Satterthwaite’s method was used to estimate degrees of freedom. The results are presented in Figure 4, and raw values are presented in Appendix 9. The classical ANOVA table with Satterthwaite’s method for the estimation of degrees of freedom is presented in Appendix 10.

These contrasts revealed that RTs were shorter for highly typical patients than for unusual ones*, t*(69.0)=3.97, *p*<0.001*, d*=−0.16 (−0.28 – 0.05). As in Experiment 1, adults responded faster than children, *t*(59.8)=4.78, *p*<0.001, *d*=0.25 (0.08 – 0.42), 10-year-old children responded marginally faster than younger ones, *t*(71.3)=1.76, *p*=0.08, *d*=0.14 (−0.03 – 0.32), and there was no difference between 6- and 8-year-old children, *t*(73.0)=1.06, *p*=0.29, *d*=0.14 (−0.13 – 0.40).

Together, these results showed that unusual patients disrupt grammatical judgments, as reflected in both accuracy and processing speed. This effect was observed in all participants (i.e., both adults and children). The fact that this disturbance is similar in all age groups suggests that it is due to cerebral structures and cognitive processes that become efficient early in the development. In the D/P framework of language (Ullman, 2001), procedural memory appears to be the best candidate to explain why the effect is similar at all ages. Indeed, procedural memory may be functional as early as 2months of age (e.g., Canfield and Haith, 1991), whereas the developmental trajectory of declarative memory is more uncertain. Some studies of declarative memory have shown that no developmental growth can be observed (e.g., Anooshian, 1999), while others suggest that this memory system continues developing until adulthood (e.g., Perruchet et al., 1995).

Nevertheless, this interpretation would be more convincing if (1) we could show that the four main analyses revealed no interaction effect, and (2) we could show that unusual items were processed as if they were ungrammatical. The first requirement can be met by using Bayes factors (e.g., Wetzels et al., 2011), which allow one to determine whether evidence exists in favor of the null hypothesis. To fulfill the second requirement, one can use Bayes factors on the results of linear mixed models for ungrammatical judgments.

#### Supplemental Analyses: Bayes Factors for the Interaction Terms

The interaction effect in the four main analyses was further analyzed using Bayes factors, obtained with bayestestR (Makowski et al., 2019). Bayes factors can be interpreted as evidence for the null hypothesis if the value is <0.33 (Wetzels et al., 2011). We tested the interaction term for Experiments 1 and 2, for both accuracy and RTs. Our results showed that, in both Experiment 1 (BF_accuracy_<0.001 and BF_RTs_<0.001) and Experiment 2 (BF_accuracy_<0.001 and BF_RTs_<0.001), there was evidence supporting the null hypothesis concerning the interaction term.

#### Supplemental Analyses: Analysis of Ungrammatical Items in Experiment 2

In order to determine whether meaningless items were considered as ungrammatical, we first performed a generalized mixed model in which responses of “ungrammatical” were considered as correct for both ungrammatical items (which is obvious) and unusual items. This choice is justified by the fact the main analysis suggested that children processed unusual patients as if they were ungrammatical. It should also determine whether children and adults behave similarly. This analysis would allow one to determine whether there were differences in the ways adults and children process items in which the patients are unusual in the context of the sentence and in the way children process items with unusual patients compared to ungrammatical items. We mimicked the main analysis, except that the levels investigated in the Type of item variable concerned ungrammatical items and unusual items for which the decision was “ungrammatical.” This choice is justified by the fact that it is more rigorous to compare the same decision instead of comparing different decisions in the analyses.

**Figure 5:**
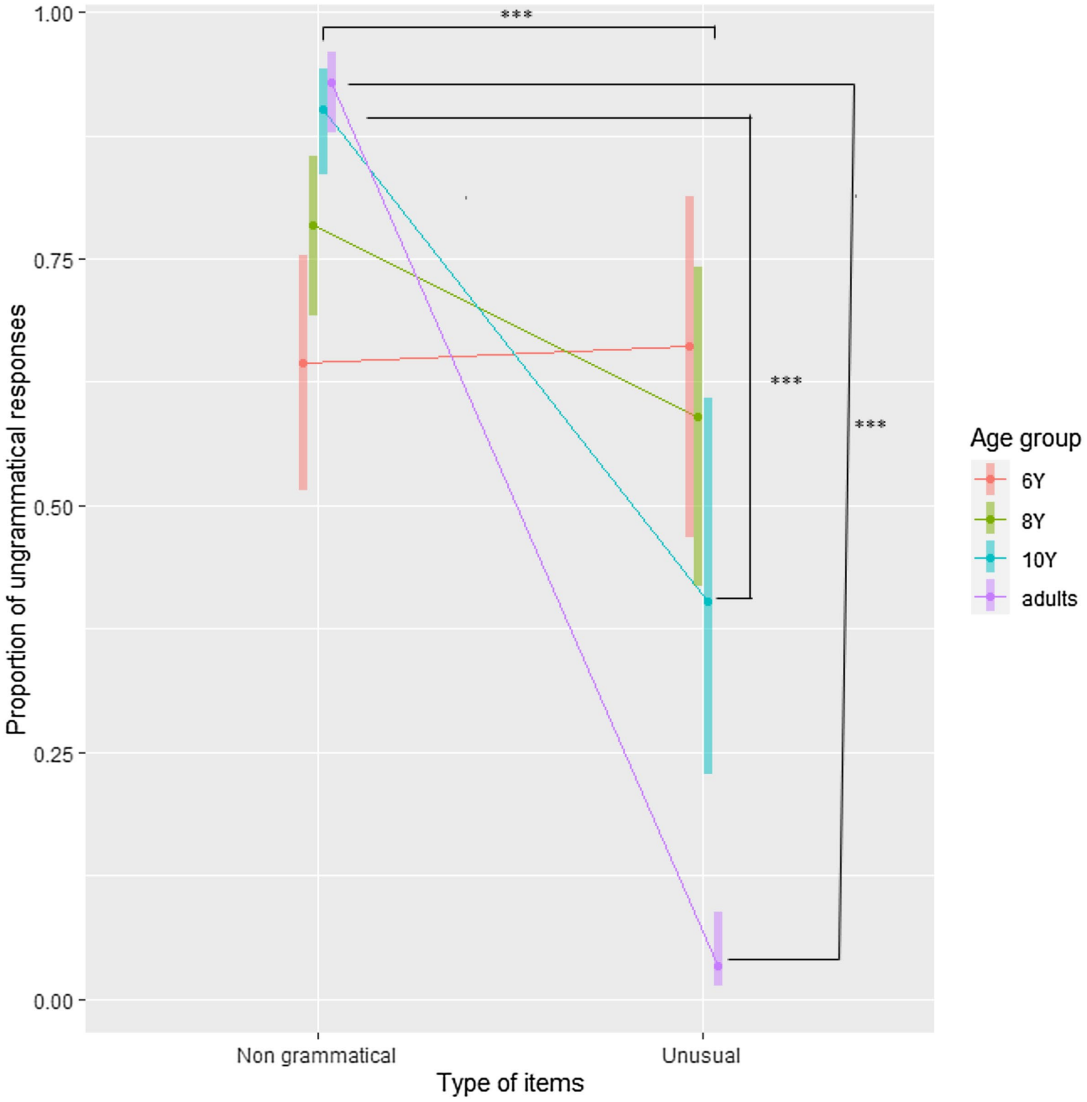
Mean accuracy results plotted separately for Age group and Type of item for “ungrammatical” responses in Experiment 2. Error bars are 95% CI. ^***^p<0.001.

These analyses revealed that ungrammatical items are more likely to be considered as ungrammatical than unusual items, *χ*(3)²=579.72, *p*<0.001, Δ*R*^2^=0.146. Nevertheless, these results could be explained by developmental stages in discriminating what is grammatical from what is not. Indeed, the interaction is also significant, *χ*(3)²=68.06, *p*<0.001, Δ*R*^2^=0.016, contrary to the effect of Age group, *χ*(3)²=6.12, *p*=0.106, Δ*R*^2^<0.001. To further explore the developmental stages in the acquisition of this ability, we performed polynomial contrasts on the interaction between Age group and Type of item. These analyses revealed that that delineating ungrammatical sentences from meaningless sentences is an ability that is acquired throughout childhood and into adulthood and may follow a linear, *z*=8.95, *p*<0.001, or quadratic relationship, *z*=2.55, *p*<0.011. The results are summarized in Figure 5. Mean accuracy responses and SEs are presented in Appendix 11.

**Figure 6:**
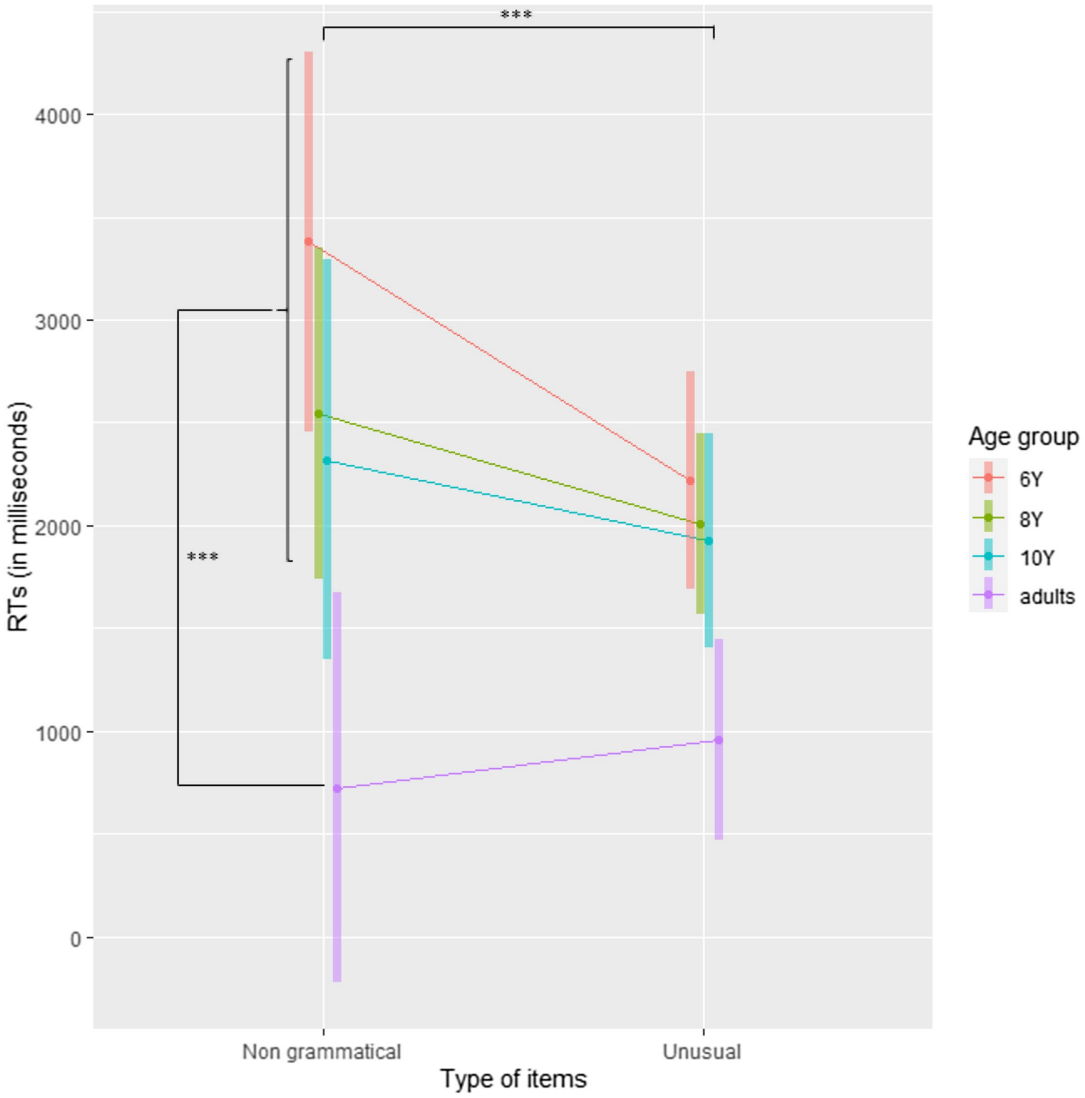
Mean RTs plotted separately for Age group and Type of item for “ungrammatical” responses in Experiment 2. Error bars are 95% CI. ^***^p<0.001.

We performed the same analysis on RTs. Twenty-five observations (i.e., <1%) were considered as outliers and removed from the analyses. Both random effects were significant and explained almost 60% of the variance. The analyses revealed that unusual items were processed faster than ungrammatical items, *χ*(3)²=1163.69, *p*<0.001, Δ*R*^2^=0.140. The effect of age was also significant, *χ*(3)²=15.81, *p*=0.001, Δ*R*^2^=0.002. However, the interaction effect was not significant, *χ*(3)²=5.13, *p*=0.16, Δ*R*^2^<0.001. The results of these analyses are summarized in Figure 6. The mean response accuracies are presented in Appendix 12.

Contrast analyses revealed that adults responded faster than children, *t*=4.43, *p*<0.001. However, 10-year-old did not respond significantly faster than younger children, *t*=1.105, *p*=0.273, and 8-year-old were no faster than 6-year-old, *t*=1.30, *p*=0.198.

## General Discussion

In this study, we wondered whether the patient typicality effect (e.g., McRae et al., 1997; Ferretti et al., 2001) could be replicated, adopting a developmental perspective in order to better understand the effect and incorporate this body of knowledge into the D/P Model (Ullman, 2001, 2004, 2020). Experiment 1 was designed to compare highly typical patients with less typical patients, while Experiment 2 compared highly typical patients with unusual patients for the particular verb. Certain results of both experiments lead us to believe that we observed a patient typicality effect.

In Experiment 1, a typicality effect was present for accuracy but not for RTs. These results contrast with those obtained by Ferretti et al. (2001), who consistently found a priming effect on RTs but not on accuracy. Different explanations can be posited to account for these discrepancies. First, given that all participants of Ferretti et al. (2001) were adults and most of our participants were children, the lack of effect could be explained by age. This explanation is coherent with the fact that the priming effect on RTs in a study of Ferretti et al. (2001) was about 30ms and that our adult group also showed a 30ms priming effect, although this effect was not significant in our study. If the absence of a priming effect on RTs is related to the presence of children in our sample, it could be because children do not present the patient typicality effect and this effect emerges after the age of 10. However, if that were the case, we should not observe a priming effect on accuracy either. Moreover, according to this explanation, we should not have seen a priming effect in Experiment 2 either. In a study of Ferretti et al. (2001), the typicality effect was observed for agents and patients that matched the thematic roles of the verb even if the frequency of production of the patient in free association norms was <0.05. In our study, less typical patients matched the thematic roles of the verb even when the frequency of production was <0.05. Thus, our results are compatible with a thematic role–based explanation: no priming effect on RTs was observed because highly and less typical patients share many thematic features.

In Experiment 2, our results showed that typical patients were processed faster and more accurately than unusual patients, and these facilitation effects were observed in all age groups. Nevertheless, even though no interaction was found, it is noteworthy that children, unlike adults, often considered meaningless sentences as ungrammatical. These relatively low accuracy rates could suggest that the grammaticality judgment task is too difficult for children. However, this task is very commonly used in the study of language acquisition (van der Lely and Ullman, 1996; Rice et al., 1999; Kail, 2004; Tremblay, 2005). For instance, Kail (2004) found that the same age groups of children were able to perform the task quite efficiently. Moreover, when all conditions are confounded, all age groups responded above the chance level. Thus, another possibility is that this task provides important information about the processes that are involved in the priming effect, from verbs to typical patients. Different possible explanations can be proposed to account for these effects, and we will integrate them into the complex relationships between declarative and procedural memory during language processing.

The first explanation of this effect involves spreading activation. According to this view, a verb is connected to nouns that represent its common agents and patients (for a more detailed explanation, see Ferretti et al., 2001). A more holistic view is that verbs integrate thematic roles, which means that, in addition to syntactic arguments, semantic features of the verb must be stored (e.g., McCawley, 1968). Our results show that the patient typicality effect cannot rely (only) on spreading activation, given that spreading activation is considered highly sensitive to free association norms and the patient typicality effect was not very sensitive to this factor in our study. Indeed, when the patient was rarely produced in free association tests but respected the verb’s thematic roles, as in Experiment 1, there was no patient typicality effect on RTs and a very small one on accuracy. Conversely, in Experiment 2, the effect was very large when the patient was not produced in free association and it did not respect the verb’s thematic roles. In other words, our results provide evidence consistent with the hypothesis about the verb’s thematic roles (McCawley, 1968; McRae et al., 1997; Ferretti et al., 2003). More specifically, our results agree with the fourth experiment in the study of Ferretti et al. (2002), which showed that syntactic structure was involved in the occurrence of the priming effect for congruent patients, and with the fact that syntactic structure is associated with a large priming effect (West and Stanovich, 1986). On the contrary, although spreading activation probably contributes, the experimental design of this study suggests that models that rely mainly on this process (e.g., MacDonald et al., 1994) do not capture important aspects of the patient typicality effect. In other words, we believe that both semantics and syntax contribute to these effects (Stanovich and West, 1983; Morris, 1994) but that our experimental design makes the syntactic aspects more prominent.

A second explanation can be found in the development of metalinguistic awareness, which refers to the ability to manipulate the structural features of language and to focus on the language form rather than the meaning (Chaney, 1992). Cairns et al. (2006) showed that younger children are able to make a grammatical judgment and identify ill-formed sentences but are not able to correct them. In this study, younger children may have identified that sentences with an unusual patient are ill-formed when it is not possible to find meaning but metalinguistic abilities are not sufficiently developed to identify that the problem is not syntactic. In other words, younger children cannot segregate the form from the meaning. This explanation follows the study of Skeide et al. (2014), who argue that semantics and syntax are intertwined during childhood and the segregation does not occur before the age of 10.

In the context of the D/P Model, several results suggest that the patient typicality effect depends on procedural memory. First, since spreading activation is less likely than the verb’s thematic roles to explain the typicality effect, and since the thematic role explanation is modulated by syntactic cues, procedural memory appears to be a good candidate to account for this effect. Moreover, developmental growth should occur in priming tasks that involve semantic memory (Kareev, 1982; MacDonald et al., 1994; Perraudin and Mounoud, 2009; Collette et al., 2016), whereas, according to the developmental invariance hypothesis (Meulemans et al., 1998; Ullman, 2020) and the idea that grammar is sustained by procedural memory (e.g., Ullman, 2001), syntactic representations should emerge early during cognitive development (Shimpi et al., 2007). Our results showed that children respond slower and less accurately than adults, but the absence of an interaction between age and item type suggests that priming occurred similarly in all age groups, for both RTs and accuracy. Thus, our results seem coherent with the developmental invariance hypothesis. Nevertheless, these results must be moderated by the fact that children responded at the chance level in the meaningless (unusual patient) condition. We suggest that, when children were unable to assign a meaning to the sentence (i.e., especially in the meaningless condition), they tended to consider the sentence to be ungrammatical. This was not the case for adults. Thus, even though the interaction was not significant, we can suspect that the processes involved in performing the task were not the same in children as in adults. If the patient typicality effect was explained by declarative memory alone, we could expect children to be less accurate in the meaningless condition than in the highly typical condition, but there was no reason to predict that accuracy should fall to the chance level. Conversely, if thematic roles are sustained by procedural memory, we would expect meaningless sentences to be considered ungrammatical. This interpretation is strengthened by the fact that there were no differences in younger children’s accuracy for ungrammatical items and meaningless items. Thus, these results suggest that, at least for children, grammar and the ability to find the meaning are closely intertwined, and that procedural memory must be involved in determining the meaning of a sentence. This view is shared by Skeide et al. (2014) who suggest that syntax sets gradually apart from semantics. Unlike children, adults can distinguish between sentences in which the grammar is correct but there is no clear meaning and sentences in which grammar is violated. This developmental pattern can be explained by the development of metalinguistic skills that depends on declarative memory (Cairns et al., 2006).

These results can be interpreted in light of the D/P Model. Indeed, Ullman and Pierpont (2005) suggested that declarative memory could compensate for the procedural deficit hypothesized to exist in specific language impairment (SLI). Moreover, the D/P Model suggests that irregular morphological forms are stored in declarative memory. Thus, a more flexible way to conceptualize how procedural and declarative memory deal with grammar is to consider that grammar may rely on procedural memory if language is used automatically (i.e., without being aware of the grammatical rules that are being applied), which occurs when language is used conventionally, but these rules come to be stored in declarative memory, for instance through education and experience with language, and can be checked in declarative memory when necessary. Typically developing individuals usually rely only on procedural memory to use grammar efficiently. However, when their grammar is explicitly tested, they must examine their declarative memory. In this study, we asked participants to make grammaticality judgments, which mean that, when participants know grammar rules explicitly, they can directly test their knowledge based on declarative memory. However, since these rules are explicitly mastered only in adulthood, when children find a sentence to be meaningless, they have difficulties deciding whether a grammatical rule has been violated or the grammar is correct but the semantics is anomalous. Our view follows Cairns et al. (2006) who consider that metalinguistic skills allow children to access syntactic knowledge consciously in order correct ungrammatical sentences. This explanation would also account for the fact that the priming effect is especially large in Experiment 2, and latencies in the meaningless condition are longer than in the ungrammatical condition. Indeed, grammatical items with highly typical patients are processed about 50ms faster than ungrammatical items, but meaningless sentences are processed about 230ms slower. We suggest that, for sentences with typical patients and sentences in which grammar is violated, participants rely only on their procedural memory to make their decisions (which does not mean that declarative memory plays no role in understanding the sentence). For sentences in which the patients are meaningless in context, procedural memory allows participants to detect that something is strange because the patient does not respect the verb’s thematic role, and declarative memory is then examined to determine whether a grammatical rule has been violated or whether it is possible to assign a meaning to the sentence (e.g., metaphorical); this process would explain why the differences in response latencies are so large.

This interpretation of our results is supported by a previous study (Drouillet et al., 2018) in which we showed that the gap between typical patients and meaningless patients was predicted by individual differences in implicit procedural memory abilities. We also showed that these individual differences predicted the ability to understand verbal metaphors, namely metaphors involving a verb and a patient (e.g., “catapulted his words”). From a mechanistic point of view, we suggest that, when we hear a sentence, procedural memory quickly decides whether the order of words, the syntax, and the thematic roles of words are respected, so that it is possible to understand the sentence. Thus, we suggest that procedural memory determines not only whether syntax and grammar are correct but also whether it is possible to comprehend the sentence. Nevertheless, since the comprehension of the sentence has not been tested directly, future research should more directly assess the role of procedural memory in understanding. More specifically, we suggest that procedural memory anticipates what kinds of words are allowed after a specific word, which relies on the word’s syntactic features but also its semantic traits. For instance, after the verb *give*, a patient is required, and this patient must be something that can be given. When the word occurs, its meaning is activated in declarative memory while procedural memory checks for irregularities and anticipates the following word. This incremental word activation allows one to comprehend the specific meaning of the sentence in question.

When grammar is violated or when semantics is anomalous (e.g., because the thematic role is not respected), the sentence is rejected by procedural memory, and declarative memory must explicitly examine it, applying two processes: an explicit check for grammar violations and an explicit search for meaning even if the sense is not obvious. This situation occurs when one is exposed to a new metaphor or complex information, as in teaching situations. In this case, the meaning must be created synchronously by exploring semantic networks through automatic (i.e., spreading activation) and controlled (i.e., expectancy generation) processes. A strong prediction made by this view is that individuals with richer semantic networks should be able to find meanings in more complex situations. We can also expect that language will be less fluent for complex ideas than for easy ones, independently of syntactic structure and word frequency. Moreover, since metaphors are often used to explain complex ideas (e.g., Weigmann, 2004), by creating links with previously acquired knowledge, we can predict that, when metaphors are used to explain complex ideas, the easier a metaphor is for an individual to understand (i.e., procedural memory does not identify any irregularities), the more helpful it will be for apprehending the ideas.

Our study has important implications for the D/P Model. First, it proposes some hypotheses about the dynamic nature of the interactions between declarative and procedural memory so that language can be used fluently and efficiently. Indeed, the D/P Model can explain what is stored in each memory system and why some redundancies are necessary, but it has not previously elaborated on how these memory systems interact when one is using language *in vivo*. Although our study is a first step in this direction for receptive language, our suggestions should also be tested for expressive language. Second, we suggest that the model should evolve so that the role of procedural memory in language is not limited to grammar (broadly speaking) but is also involved in meaning. More specifically, in accordance with Ullman (2020), who suggests that procedural memory may play a role in predicting the next word in a sentence (for a similar view, see Chang et al., 2006), we believe that procedural memory plays a role in determining a patient’s compatibility with a verb. However, we also believe that procedural memory must be involved in the processing of meaning, contrary to Ullman. Indeed, Ullman’s view cannot explain why less typical patients do not slow down grammatical decision latencies (i.e., if the meaning of a verb–patient pair depends on declarative memory, we should have obtained a frequency effect, which is not the case), whereas meaningless patients have an effect that is five times greater than the difference between highly typical patients and ungrammatical items. Nor does it explain why individual differences in implicit procedural learning are related to the difference between typical and meaningless patients (Drouillet et al., 2018).

Strong predictions can be made on the basis of our suggestion. First, according to the Procedural Deficit Hypothesis (Ullman and Pierpont, 2005), children with SLI have impaired procedural memory, they should make more mistakes in a grammaticality judgment task than typically developing children, especially for inflectional operations (Sengottuvel and Rao, 2013), and the gap between typical patients and meaningless patients in the context of the sentence should be less pronounced for children with SLI than for their typically developing peers. Second, if the task that participants have to perform involves semantic judgment (e.g., animacy judgment and lexical decision), the disruption caused by meaningless patients should be less pronounced and accuracy should be above the chance level for all age groups. Third, for a given age group, the accuracy gap between typical and meaningless patients should be inversely related to explicit mastery of grammar rules.

One limitation on this study is the fact that we explored the patient typicality effect but not the agent typicality effect. Although we have no reason to think that the processes involved in processing agents differ from those involved in processing patients, this hypothesis must be tested. Related to this limitation, in our study, the patient was consistently presented at the end of the sentence, whereas grammatical errors can occur anywhere in a sentence. Thus, it is not always necessary to hear a whole sentence before judging its grammaticality. A solution to this issue would be to use passive forms: although passive structures are more difficult for children to understand, the position of the patient would ensure its correct processing. This study also does not allow participants to correctly apprehend situations in which meaning is possible but must be constructed synchronously, as is the case for new metaphors.

To the best of our knowledge, this study is the first to integrate studies of the patient typicality effect within a broader model of language, namely the Declarative/Procedural Model. Our proposal provides a more dynamic framework for language processing and proposes that procedural memory has additional roles that had not previously been considered. Our proposal makes strong, testable predictions and responds to an important limitation of the model that had not been addressed much before: how does declarative and procedural memory interact to allow the fluent use of language?

## Data Availability Statement

Data are available in Supplementary Material and on OSF: https://osf.io/4znu9/.

## Ethics Statement

Ethical review and approval was not required for the study on human participants in accordance with the local legislation and institutional requirements. Written informed consent to participate in this study was provided by the participants’ legal guardian/next of kin.

## Author Contributions

CD, VB, and NS conceived the experiment, the material, and the procedure, read the entire manuscript, provided insights on all the sections, and agreed on the final form of the manuscript. CD and NS wrote the abstract. CD wrote the introduction. NS wrote the method, the results, and the discussion. All authors contributed to the article and approved the submitted version.

## Conflict of Interest

The authors declare that the research was conducted in the absence of any commercial or financial relationships that could be construed as a potential conflict of interest.

## Publisher’s Note

All claims expressed in this article are solely those of the authors and do not necessarily represent those of their affiliated organizations, or those of the publisher, the editors and the reviewers. Any product that may be evaluated in this article, or claim that may be made by its manufacturer, is not guaranteed or endorsed by the publisher.
