# Supplementary material for: Processing Verb Meanings and the Declarative/Procedural Model: A Developmental Study
Source: Front Psychol. 2021 Sep 30;12:714523. doi: 10.3389/fpsyg.2021.714523 (PMC8514706; doi:10.3389/fpsyg.2021.714523)
Supplement: Supplementary file 1 [file Data_Sheet_1.PDF]

APPENDIX 1

Table S1. Verb and their three possible patients (and their translation)

Verb	Typical patient	Less typical patient	Unusual patient
Arroser (Water)	fleurs (flowers)	sœur (sister)	chance (luck)
Colorier (Colour)	dessin (drawing)	visage (face)	poussière (dust)
Construire (Build)	maison (House)	boîte (box)	corps (body)
Creuser (Dig)	trou (hole)	sol (ground)	danse (dance)
Déchirer (Tear)	feuille (leaf)	robe (dress)	ferme (farm)
Enfoncer (Press in)	clou (nail)	bâton (stick)	barbe (beard)
Oublier (Forget)	clé (key)	rêve (dream)	boue (mud)
Plier (Fold)	feuille (leaf)	robe (dress)	vague (wave)
Réparer (Repair)	voiture (car)	collier (necklace)	pensée (thought)
Sécher (Dry)	cheveux (hair)	habit (habit)	secret (secret)
Vider (Empty)	poubelle (trash can)	tiroir (drawer)	virgule (comma)
Eclairer (Enlighten)	pièce (room)	livre (book)	fonction (function)
Effacer (Wipe off)	tableau (board)	texte (text)	hasard (hazard)
Envoyer (Send)	lettre (letter)	argent (money)	rôle (role)
Eteindre (Switch off)	lumière (light)	moteur (engine)	milieu (middle)
Libérer (Release)	prisonnier (prisoner)	élève (prisoner)	accident (accident)
Renverser (Overturn)	verre (glass)	table (table)	science (science)

Salir (Dirty)	vêtements (clothes)	toilette (outfit)	surprise (surprise)

APPENDIX 2

Table S2. Sentences used in the task

Condition	Item (translation) with their typical/less typical/unusual patient. Grammatical violations are in bold
**Training**	Jusqu'au soir, en passant par différentes pistes, descend le skieur la montagne.<br><br>(Until the evening, passing through various tracks, **skies down the skier** the mountain)
	Dans la rue, le gendarme poursuivent les bandits pour récupérer l'argent volé.<br><br>(In the street, the police officer **pursue** the bandits to recover the stolen money.)
	Dans les bras de sa mère, le bébé s'endort doucement en écoutant musique la.<br><br>(In his mother's arms, the baby gently falls asleep listening to music **the**).
	En sortant du hangar, avant de grimper dans son avion, le pilote tiennent son casque.<br><br>**(**When leaving the hangar, before climbing into his plane, the pilot **hold** his helmet)
	En arrivant dans l'atelier, avant de recevoir le mannequin, le photographe installe son matériel.<br><br>(Upon arriving in the studio, before receiving the model, the photographer sets up his equipment.)
	Sur la plage, avant de lâcher le cerf-volant, le père vérifie les cordes.

	(On the beach, before releasing the kite, the father checks the ropes.)
	Après son goûter d'anniversaire, lorsque ses amis sont partis, l'enfant range sa chambre. **(**After his birthday snack, when his friends are gone, the child tidies up his room**)**
	En regardant le calendrier accroché en face de lui, le journaliste organise son voyage. **(**Looking at the calendar hanging in front of him, the journalist organizes his trip.**)**
**Target items**	Ce matin, tandis que les nuages s'éloignent dans le ciel, le soleil éclaire la pièce/le livre/la fonction (This morning, as the clouds move away in the sky, the sun shines on the room / the book / the function)
	A chaque fois qu'elle n'a plus de place pour écrire, la maîtresse efface le tableau/le texte/ le hasard (Whenever there is no more room to write, the teacher erases the board / the text / the chance)
	Aujourd'hui, pour faire plaisir à leurs enfants partis en colonie de vacances, les parents leur envoient des lettres/de l'argent/des rôles (Today, to please their children who have gone to summer camp, parents send them letters / money / roles)
	Dans l'appartement, alors que ses maîtres sont partis, le chien vide la poubelle/le tiroir/la virgule (In the apartment, while his owners are gone, the dog empties the trash / the drawer / the comma)
	Dans la boutique, pendant que la patronne reçoit les clients, l'employée sèche les cheveux/les habits/les secrets (In the shop, while the boss receives customers, the employee dries hair / clothes / secrets)

En chahutant dans le jardin près de la mare, la jeune fille salit ses vêtements/sa toilette/sa surprise (Heckling in the garden near the pond, the young girl dirties her clothes / her toilet / her surprise)
Habituellement, dès que le directeur lui en donne l'autorisation, le surveillant libère les prisonnier/les enfants/les accidents (Usually, as soon as the director gives permission, the warden releases prisoners / children / accidents)<br>NB: In French,,the word "warden" and "supervisor" are homonym.
Le plus souvent possible, pour économiser de l'énergie, la femme éteint la lumière/le moteur/la science (As often as possible, to save energy, the woman turns off the light / engine / science)
Pendant son numéro, pour faire rire les enfants, le clown renverse son verre/la table /la science (During his act, to make the children laugh, the clown spills his glass / the table / science**)**
Après avoir raccroché le téléphone, l'air songeur, la femme plie la feuille/la robe/la vague (After hanging up the phone, thoughtfully, the woman folds the sheet / the dress / the wave)
Ce soir de fête, avant de rejoindre sa famille, l'artisan répare la voiture/le collier/la pensée. (This festive evening, before joining his family, the craftsman repairs the car / the necklace / the thought)
Dans le terrain vague à côté des nouveaux immeubles, les ouvriers creusent un trou/le sol/la danse (In the vacant lot next to the new buildings, the workers dig a hole / the ground / the dance)
En été, lorsqu'il fait chaud, le petit garçon arrose les fleurs/sa sœur/la chance<br>**(**In summer, when it is hot, the little boy waters the flowers / his sister / luck)

	Parfois, en partant marcher dans la campagne, le promeneur oublie sa clé/son rêve/sa boue (Sometimes, when going for a walk in the countryside, the walker forgets his key / his dream / his mud)
	Pour aménager la barrière, pendant que son apprenti tient les outils, le menuisier enfonce le clou/le bâton/la barbe<br><br>(To arrange the barrier, while his apprentice holds the tools, the carpenter presses in the nail / stick / beard)
	Régulièrement, si elle se presse de trop en traversant les couloirs, la journaliste déchire sa feuille/sa robe/sa ferme<br><br>(Regularly, if she hurries too much while crossing the corridors, the journalist tears her sheet / her dress / her farm)
	In his notebook, while the other children are at recess, the sick pupil colors a drawing / a face / a dust
	Tous les jours, lorsqu'il va au travail, l'homme construit des maisons/des boîtes/des corps<br><br>**(**Every day when he goes to work, the man builds houses / boxes / bodies)
**Sentence with grammatical violation**	Sur l'île noire, après avoir coulé le bateau, bandit le enfouit le trésor<br><br>**(**On the black island, after sinking the boat, bandit **the** buries the treasure)
	Très souvent, sur un petit carnet personnel, cliente la inscrit les achats<br><br>(Very often, on a small personal notebook, customer **the** records the purchases)

	Aux frontières, après avoir vérifié les passeports, douanier le défait les valises<br><br>(At the border, after checking the passports, customs **the** officer unzips the suitcases)
	En ville, pharmacienne la soigne les dames avec des produits provenant de chine<br><br>**(**In town, pharmacist **the** treats the ladies with products from China)
	A Noël, avant de décorer le magasin, le patron garnit sapin le<br><br>**(**At Christmas, before decorating the store, the boss garnishes Christmas tree **the**)
	Certaines fois, pour obtenir le silence dans la classe, la maîtresse punit élèves les<br><br>(Sometimes, to obtain silence in the classroom, the teacher punishes pupils the)
	Aujourd’hui, pour fêter la réussite du concert, la chanteuse reçoit bouquet un<br><br>**(**Today, to celebrate the success of the concert, the singer receives bouquet **a**)
	Le mardi, la marchande fournit poisson du à tous les habitants du village<br><br>**(**On Tuesday, the merchant supplies fish **some** to all the inhabitants of the village)
	Depuis hier, le facteur transmet courrier le au volant d’une fourgonnette jaune<br><br>(Since yesterday, the postman is bringing mail the on the wheel of a yellow van)
	Tous les soirs, jusqu’à la tombée complète de la nuit, relit la comtesse des poèmes<br><br>Every evening, until nightfall, **rereads the countess** of the poems
	Tous les mois, afin de gagner beaucoup d’argent, revend le voleur des bijoux

	**(**Every month, in order to earn a lot of money, **sell the jewelry** thief)
	A l'église, bénit le curé les mariés après avoir célébré la messe<br><br>**(**In church, **blesses the priest** the bride and groom after having celebrated mass
	Vers 7 heures, prévient la vendeuse les clients avant de fermer le magasin<br><br>**(**Around 7 a.m., **warns the saleswoman** the customers before closing the store**)**
	Chaque dimanche, avant de recevoir des invités, la princesse le château décore<br><br>**(**Every Sunday, before receiving guests, the princess **the castle decorates**)
	Chaque année, très haut dans les montagnes du pays, la bergère les moutons conduit<br><br>**(**Every year, very high in the mountains of the country, the shepherdess **the sheep leads**)
	Chaque jeudi, pour tous les commerçants de la ville, la banquière des factures écrit<br><br>**(**Every Thursday, for all the traders in the city, the banker **bills writes)**
	Tous les ans, le nageur une médaille obtient pour l'exécution du meilleur plongeon<br><br>(Every year, the swimmer **a medal obtains** for the execution of the best dive)
	Chaque week-end, en faisant des exercices à la barre, la danseuse un tutu salit<br><br>(Every weekend, doing exercises on the barre, the dancer **a tutu dirties**)
	Dans le stade, en courant le plus rapidement possible, le sportive franchit les obstacles<br><br>(In the stadium, running as quickly as possible, **the athlete** overcomes obstacles)

	Nb: in French, ‘sportive’ is a woman and should be preceded by “la” instead of “le”.
	Le matin, avant de s’envoler dans les airs, le sorcière saisit un balai<br><br>**(**In the morning, before flying into the air, **the witch** grabs a broom)<br><br>Nb: in French, ‘sorcière is a woman and should be preceded by “la” instead of “le”.
	Pour dîner, le cousine farcit le poulet après avoir préparé les entrées<br><br>(For dinner, **the cousin** stuffs the chicken after preparing the starters)<br><br>Nb: in French, ‘cousine is a woman and should be preceded by “la” instead of “le”.
	Fréquemment, en racontant de très longues histoires, la papy endort les enfants<br><br>**(**Often, by telling very long stories, **the grandpa** puts the children to sleep)<br><br>Nb: in French, ‘papy is a man and should be preceded by “le” instead of “la”.
	Sans pitié, la chasseur abat le gibier avec une grande carabine à plombs<br><br>**(**Mercilessly, **the hunter** slaughters game with a large pellet rifle)<br><br>Nb: in French, ‘chasseur’ is a man and should be preceded by “le” instead of “la”.
	En été, la pêcheur rejoint le rivage une fois le bateau rempli de poissons<br><br>(In summer, **the fisherman** reaches the shore once the boat is full of fish)<br><br>Nb: in French, ‘pêcheur is a man and should be preceded by “le” instead of “la”.
	En hiver, dans la salle de gymnastique, la fillette suspendent les cerceaux<br><br>(In winter, in the gymnastics hall, **the girl hang** the hoops**)**

	A midi, dans la meilleure pâtisserie de la ville, la serveuse choisissent un gâteau<br><br>(At noon, in the best pastry shop in town, **the waitress choose** a cake)
	De nos jours, grâce aux progrès des recherches médicales, le docteur guérissent les malades<br><br>(Nowadays, thanks to the progress of medical research, **the doctor heal** the sick)
	Le lundi, la lingère blanchissent les mouchoirs en utilisant un nouveau produit<br><br>(On Monday, **the maid whiten** the handkerchiefs using a new product)
	Dans le théâtre, grâce à l'aide de tous les ouvriers, le décorateur peignent la nouvelle scène<br><br>(In the theater, with the help of all the workers, **the decorator paint** the new scene)
	A Paris, le boxeur surprennent le public en gagnant contre le champion du monde<br><br>(In Paris, **the boxer surprise** the public by winning against the world champion)
	Chaque été, avec du sable et des coquillages, le garçon bâtit une château<br><br>(Every summer the boy builds **a castle** with sand and shells)<br><br>NB: in French, "château" is masculine and should be preceded by "un" instead of "une"
	Chaque semaine, après avoir fait les courses au marché, la voisine remplit la frigo<br><br>(Every week, after shopping at the market, the neighbor fills **the fridge**)<br><br>NB: in French, "frigo" is masculine and should be preceded by "le" instead of "la"

	Quelques fois, par peur de perdre la compétition, la championne vomit la repas<br><br>**(**Sometimes, for fear of losing the competition, the champion vomits **the meal**)<br><br>NB: in French, “repas” is masculine and should be preceded by “le” instead of “la”
	Dans le pré, après avoir nettoyé la roulotte, la gitane étend la tapis<br><br>(In the meadow, after having cleaned the trailer, the gypsy spreads **the carpet**)<br><br>NB: in French, “tapis” is masculine and should be preceded by “le” instead of “la”
	Sur la piste, pendant la dernière partie de la course, le coureur ressentent une douleur<br><br>(On the track, during the last part of the race, the runner **feel a pain**)
	De bonne heure, après avoir récolté de l’avoine, la fermière nourrissent la chevaux<br><br>Early, after harvesting oats, **the farmer feed** the horses

APPENDIX 3

Figure S1. Representation of distribution of item as a random effect in Experiment 1.

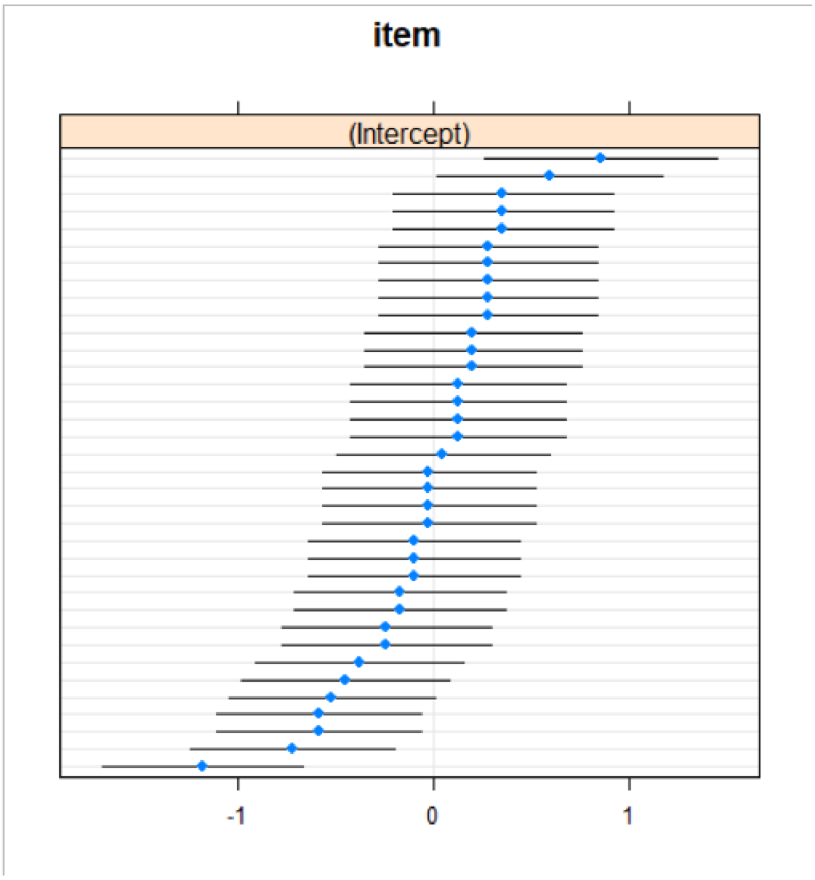

Figure S2. Representation of distribution of participants as a random effect in Experiment 1.

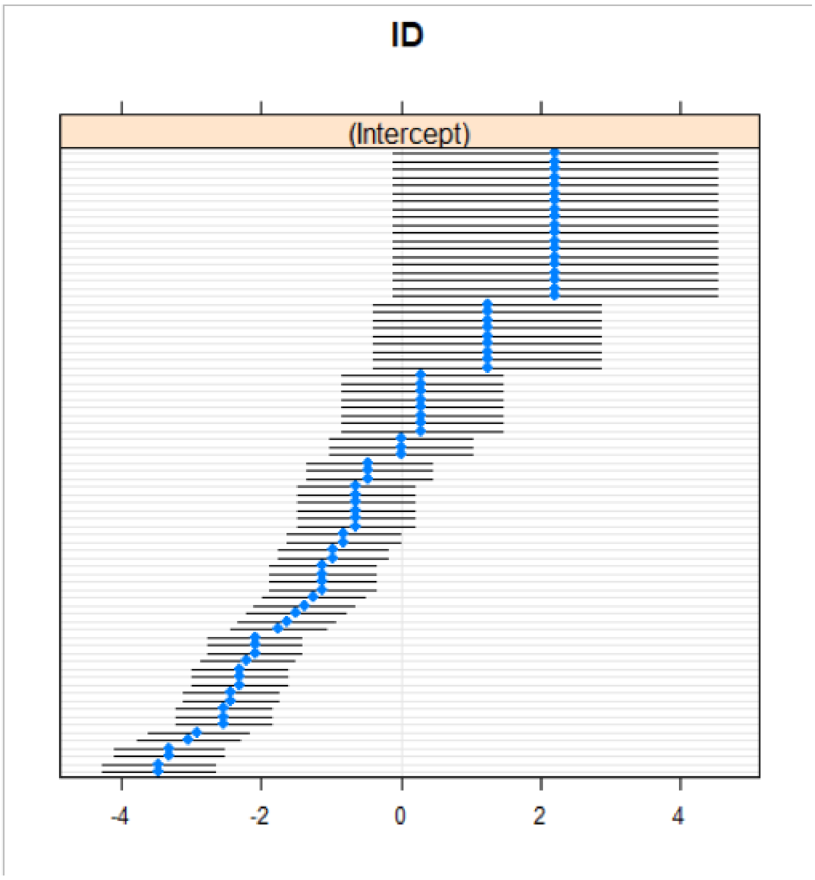

APPENDIX 4

Table S3. Probability of correct responses (and standard errors) by Age groups and Type if items

Age groups	Less Typical	Highly Typical
6Y	0.52 (0.08)	0.61 (0.09)
8Y	0.67 (0.07)	0.75 (0.07)
10Y	0.89 (0.03)	0.95 (0.02)
Adults	0.99 (<.01)	>.99 (<.01)

APPENDIX 5

Table S4. Mean RTs (and standard errors) by Age groups and Type if items

Age groups	Little Typical	Highly Typical
6Y	2436.62 (240.14)	2426.43 (246.44)
8Y	2041 (240.61)	2034.78 (247.51)
10Y	1202 (210.86)	1186.1 (216.62)
Adults	580.1 (231.41)	551.55 (237.81)

APPENDIX 6

Table S5. Classical table of ANOVA obtained by linear mixed estimation using Satterthwaite's method for the estimation of degrees of freedom for analyses of RTs in Experiment 1.

	Degrees of freedom	F value	p value
Type of items	1, 35.85	0.13	.717
Age group	3, 72.32	12.75	<.001
Type of items × Age group	3, 53.68	0.04	>.99

APPENDIX 7

Figure S3. Representation of distribution of item as a random effect in Experiment 2.

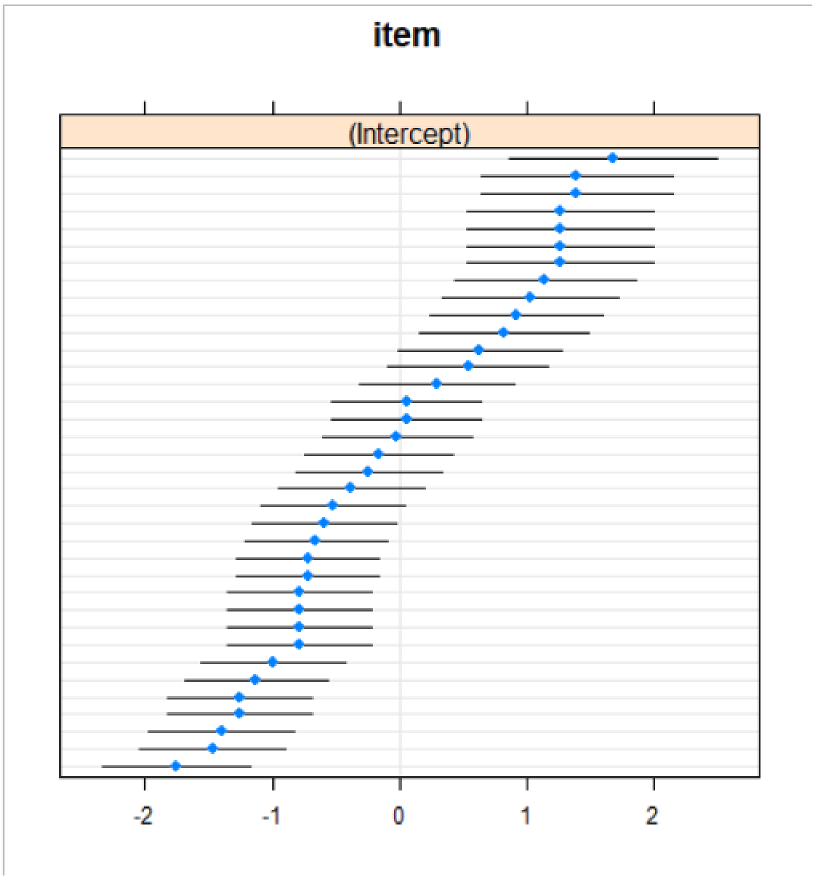

Figure S4. Representation of distribution of participants as a random effect in Experiment 2.

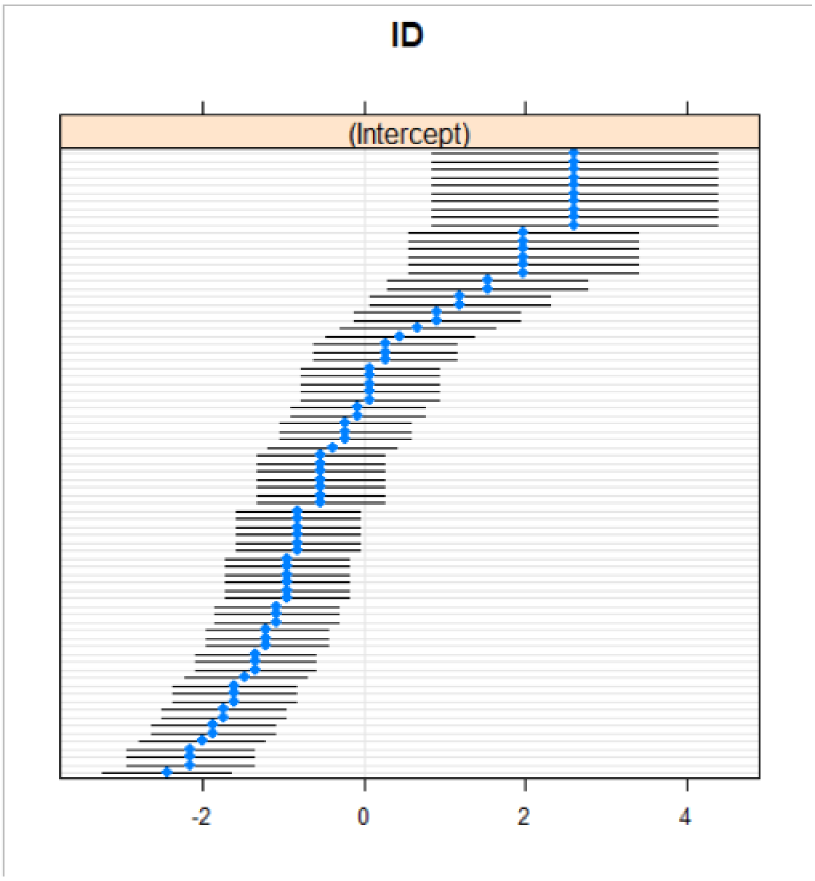

APPENDIX 8

Table S6. Mean accuracies (and standard errors) by Age groups and Type if items

Age groups	Unusual	Highly Typical
6Y	0.34 (0.09)	0.79 (0.06)
8Y	0.43 (0.08)	0.86 (0.04)
10Y	0.59 (0.1)	0.92 (0.03)
Adults	0.97 (0.02)	> 0.99 (<0.01)

APPENDIX 9

Table S7. Mean RTs (and standard errors) by Age groups and Type if items

Age groups	Unusual	Highly Typical
6Y	2224.4 (264.55)	2082.2 (178.92)
8Y	2127.44 (225.5)	1594.98 (157.11)
10Y	1796.97 (271.36)	1310.74 (187.24)
Adults	901.3 (257.37)	669.57 (181.01)

APPENDIX 10

Table S8. Classical table of ANOVA obtained by linear mixed estimation using Satterthwaite's method for the estimation of degrees of freedom for analyses of RTs in Experiment 2.

	Degrees of freedom	F value	p value
Type of items	1, 68.97	15.77	<.001
Age group	3, 70.96	8.14	<.001
Type of items × Age group	3, 55.97	1.69	.180

APPENDIX 11

Table S9. Mean accuracies (and standard errors) by Age groups and Type if items

Age groups	Non-grammatical	Unusual
6Y	0.64 (0.06)	0.66 (0.09)
8Y	0.78 (0.04)	0.59 (0.09)
10Y	0.9 (0.03)	0.4 (0.1)
Adults	0.93 (0.02)	0.03 (0.02)

APPENDIX 12

Table S10. Mean RTs (and standard errors) by Age groups and Type if items

Age groups	Non-grammatical	Unusual
6Y	3381.37 (463.96)	2216.93 (266.03)
8Y	2541.25 (404.64)	2009.64 (220.39)
10Y	2319.56 (489.47)	1922.23 (261.45)
Adults	722.62 (475.7)	954.3 (244.32)
